# Cannabizetol, a Novel Cannabinoid: Chemical Synthesis, Anti-inflammatory Activity and Extraction from *Cannabis sativa* L

**DOI:** 10.1021/acs.jnatprod.5c00826

**Published:** 2025-09-25

**Authors:** Luca Pozzi, Andrea Gotti, Marco Fumagalli, Andrea Citarella, Valerio Fasano, Giuseppe Paladino, Umberto Ciriello, Salvatore Princiotto, Francesca Annunziata, Giulia Martinelli, Enrico Sangiovanni, Andrea Pinto, Mario Dell’Agli, Daniele Passarella

**Affiliations:** † Department of Chemistry, 9304 Università degli Studi di Milano, 20133 Milan, Italy; ‡ Department of Food, Environmental and Nutritional Sciences (DeFENS), 9304 Università degli Studi di Milano, 20133 Milan, Italy; § Department of Pharmacological and Biomolecular Sciences “Rodolfo Paoletti”, 9304 Università degli Studi di Milano, 20133 Milan, Italy; ∥ 35512 LINNEA SA, 6595 Riazzino, Ticino, Switzerland

## Abstract

We report the first isolation of a previously unknown cannabinoid, cannabizetol (CBGD, **7**), from *Cannabis sativa* extracts, representing the third member of the rare class of methylene-bridged dimeric cannabinoids. The availability of a chemically synthesized standard was crucial for its unequivocal identification, thus confirming the natural occurrence of this new compound. In addition to this structural discovery, we demonstrate that cannabizetol exhibits remarkable antioxidant and skin anti-inflammatory activity, significantly higher than that observed for the known dimeric cannabinoid cannabitwinol (CBDD, **6**). These results highlight cannabizetol as a promising bioactive metabolite with potential dermatological applications. To further enable its study, we developed a continuous flow approach to optimize the preparation of these dimers, achieving a substantial reduction in reaction times.

## Graphical abstract

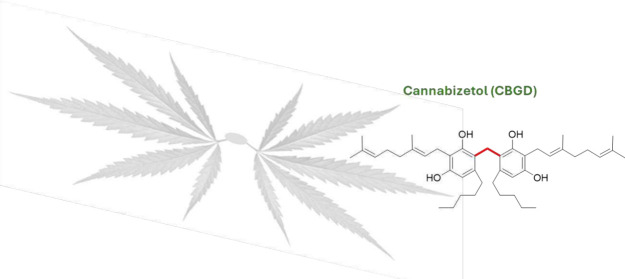

*Cannabis sativa* has been used since ancient times for recreational, ornamental, industrial, and medicinal chemistry purposes.^1^ From this plant, more than 500 compounds have been isolated to date, of which around 150 belong to the class of phytocannabinoids.^2^ Among them, Δ9-tetrahydrocannabinol (Δ^9^-THC, **1**), cannabidiol (CBD, **2**), cannabigerol (CBG, **3**) and cannabichromene (CBC, **4**) (Figure 1) are often referred to as the “major cannabinoids” because they can occur in relatively higher amounts in some *Cannabis sativa* varieties, mostly in their corresponding carboxylic acid forms. However, their abundance is highly chemovar-dependent, and in many cases these compounds may be present only at low levels; all other cannabinoids are generally grouped under the term “minor cannabinoids” due to their typically low concentrations in plant extracts.^2,3^

**Figure 1:**
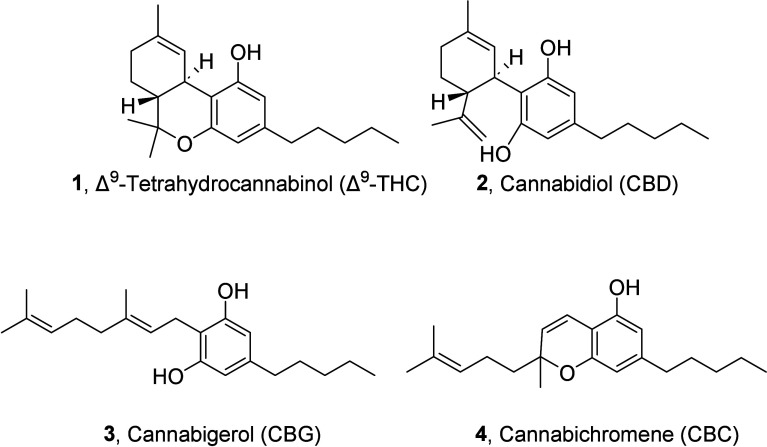
Chemical structure of Δ^9^-THC, CBD, CBG, and CBC.

Several cannabinoids have demonstrated biological activities, making *Cannabis sativa* particularly attractive as a source of potential medicinal active principles.^2^ The most potent psychoactive compound in cannabis is Δ^9^-THC, whose agonist activity on the Cannabinoid-1 (CB1) and Cannabinoid-2 (CB2) receptors of the endocannabinoid system leads to the well-known effects of marijuana, such as narcolepsy, mood and cognitive changes, increased appetite, and pain reduction.^4–6^ In general, research on the biological activity of Δ^9^-THC, CBD, CBG, and CBC is highly advanced due to their good availability through isolation or synthesis.^2^ This has led, for example, to the pharmaceutical use of a 1:1 mixture of Δ^9^-THC and CBD (marketed as Sativex^7,8^ ) for the treatment of multiple sclerosis spasticity and rare genetic forms of epilepsy (marketed as Epidiolex^9^). On the other hand, there is limited information on the bioactivity of “minor cannabinoids” due to their low quantities in plant and difficult isolation.^2^ The cannabinome^2^ has been extensively explored, leading to the discovery of numerous molecules belonging to this family; however, many cannabinoids remain unidentified and uncharacterized, mostly due to the low amount occurring in the natural matrix, the presence of complex mixtures and the subsequent difficult isolation. Nonetheless, their biological activity could be highly interesting and deserve further investigation.^2^ In fact, many “minor cannabinoids” are not biogenic products of the plant, but artifacts formed during the treatments involving the presence of heat, light, oxygen, etc. For example, cannabinol (CBN) results from the air-induced oxidative aromatization of Δ^9^-THC, while cannabicyclol (CBL) forms when CBC is exposed to natural light.^3^ These reactions result in a wide structural variety of cannabinome.^3^ For this reason, having well-characterized synthetic standards with known structures facilitates the recognition of new cannabinoids in cannabis extracts. To date, it is unusual to identify new cannabinoids that have never been previously reported. However, recent efforts in the finding, isolation, and identification of new minor cannabinoids from *Cannabis sativa* extracts led to the discovery of two new compounds: cannabisol (**5**)^10^ and cannabitwinol (CBDD, **6**)^11^ (Figure 2). These two molecules have a unique structural characteristic compared to other previously known phytocannabinoids: they are dimeric compounds, respectively of Δ^9^-THC and CBD, whose monomers are connected by a methylene bridge.

**Figure 2:**
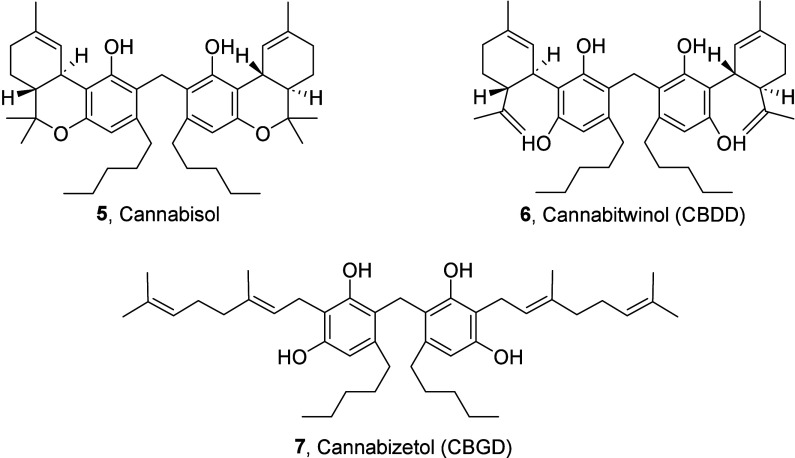
Chemical structure of cannabisol, cannabitwinol (CBDD) ,and cannabizetol (CBGD).

The serendipitous synthesis of cannabitwinol (**6**) was indeed reported as part of a medicinal chemistry effort.^12^ According to the literature, the existence of other methylene-linked dimeric natural products is well documented, and for many of them the synthesis has been described.^13^ Natural dimeric compounds are of considerable importance, as they enable further exploration of chemical space, potentially leading to novel biological activities beyond those of their respective monomers.^14^ Following our interest in cannabinoids,^15–18^ in this work we expand the pool of cannabinoid dimers by synthesizing the methylene-bridged dimer of CBG (CBGD, **7**). We decided to synthesize it based on the biosynthesis of cannabinoids, where cannabigerolic acid (CBGA) serves as the precursor to all cannabinoids. Given CBGA’s significant presence in the plant, we hypothesized that the mechanisms responsible for the formation of dimers **5** and **6**, whether biogenic or artifacts, could also apply to CBGA, potentially leading to the presence of its dimer in cannabis extracts. Two processes were followed in parallel: the synthesis of CBG dimer and the search for it in the cannabis raw material. We successfully synthesized and well-characterized dimeric compound **7** (Figure 2), which proved crucial as a reference standard for its identification and isolation from *Cannabis sativa* extracts. The dimer was then purified, characterized and compared with the synthetic compound **7**, confirming its presence in hemp extracts. In this way we found a new cannabinoid, which we decided to name “cannabizetol” inspired by Zethus, the twin brother of Amphion, son of Zeus and Antiope. He was known for his physical strength and practical skills. Additionally, based on recent findings regarding the inhibition of nuclear factor-kappa B (NF-κB) signaling in various skin cells by CBD^19^ and CBG^18^ and their derivatives, we studied the cytotoxicity and inhibition of the NF-κB driven transcription and IL-8 release of cannabizetol (**7**) and cannabitwinol (**6**) in HaCaT cells, demonstrating high efficacy for both, remarkably potent for dimer **7**. The significant biological activity of these dimeric cannabinoids prompted us to optimize the synthetic approach by exploiting the flow chemistry technology.

## Result and Discussion

### Synthesis of Methylene-Bridged CBG Dimer Cannabizetol

Based on recent reported results,^12^ CBD (**2**) was reacted with an excess of formaldehyde in 1:3 molar ratio (6 equiv) under reflux (78 °C) in EtOH for 48 h, successfully obtaining **6**, although in low yield. Isolation of the desired dimer was confirmed by comparison with spectroscopic data reported in the literature,^11,12^ confirming the validity of the procedure. We then replicated the same reaction on CBG (**3**), reacting it with 6 equiv of formaldehyde under reflux (78 °C) in EtOH for 48 h, successfully obtaining the desired CBG dimer **7**, in low yield (13%) (Scheme 1, step a). We performed a second attempt by reacting CBG with 6 equiv of formaldehyde under reflux (60 °C) in MeOH for 6 days, successfully obtaining **7** in a cleaner way but again in low yield (19%).

**Scheme 1:**
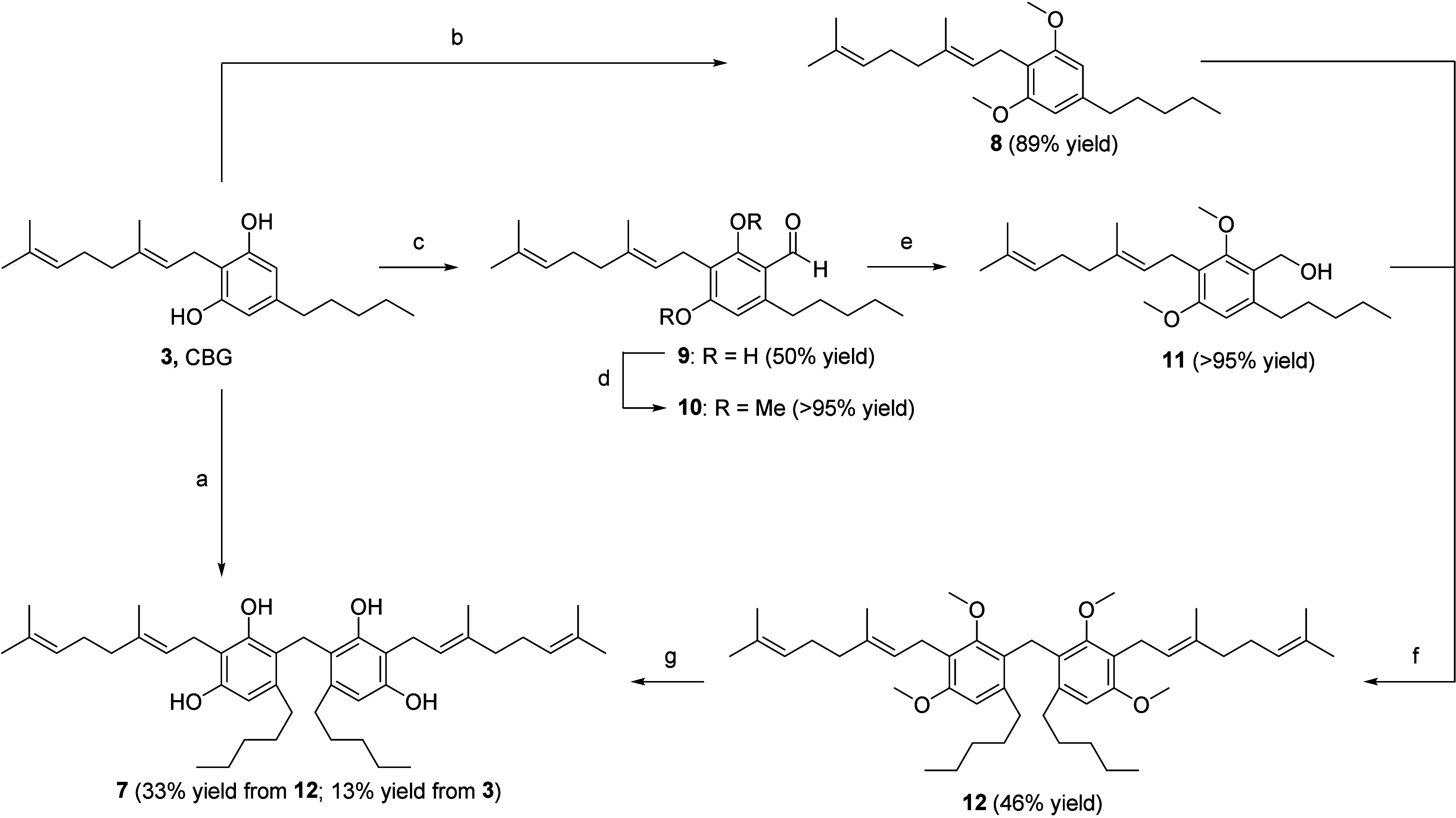
Synthesis of Cannabizetol **7** ^a^

a Reagents and conditions: (a) CH_2_O, EtOH, 78 °C, 48 h; (b) CH_3_I, Cs_2_CO_3_, dry CH_3_CN, rt to 80 °C, 5 h; (c) POCl_3_, dry DMF, 0 °C to rt, 21 h; (d) CH_3_I, Cs_2_CO_3_, dry CH_3_CN, rt to 80 °C, 2 h; (e) NaBH_4_, dry MeOH, 0 °C, 2 h; (f) *p*-TSA, dry CH_2_Cl_2_, 0 °C, 2 h; (g) (i) pentamethyldisiloxane, BCF, dry *n*-heptane, rt, Ar atmosphere, 14 h, (ii) TBAF (1 M in THF), dry THF, 0 °C to rt, Ar atmosphere, 1 h.

A synthetic pathway for cannabizetol (**7**), depicted in Scheme 1, has been developed with the purpose of finding an alternative synthesis to the formaldehyde-promoted reaction. CBG (**3**) was converted into aldehyde **9** through a Vilsmeier–Haack formylation using POCl_3_ in DMF. Aldehyde **9** was then first methylated with iodomethane and Cs_2_CO_3_ to avoid undesired intramolecular cyclization during the next reaction with *p*-TSA. Compound **10** was further reduced to benzyl alcohol **11** in the presence of an excess of NaBH_4_. CBG (**3**) was methylated with iodomethane and Cs_2_CO_3_ providing intermediate **8** in high yields. A Friedel–Crafts alkylation between intermediates **8** and **11** in the presence of *p*-TSA provided the methylated dimer **12** in moderate yields. Finally, following a strategy reported in the literature^20^ for the deprotection of the methoxy group in a different cannabinoid substrate, dimer **12** was demethylated through a Piers–Rubinsztajn reaction: by first reacting with pentamethyldisiloxane and B­(C_6_F_5_)_3_ (BCF) as a catalyst, followed by treatment of the crude with an excess of TBAF, cannabizetol (**7**) was successfully obtained. These synthetic efforts enabled the preparation and spectroscopic characterization of pure compound **7**, which will serve as a reference standard.

### Cannabizetol and Cannabitwinol Synthesis in Flow System

The low yield obtained for the synthesis of compound **7** prompted us to investigate a possible synthetic strategy using flow chemistry technology and then extend it to the synthesis of dimer **6** as well. The possibility to pressurize the reactors given by continuous systems allowed to overcome the limitation given by the boiling point of the solvent in batch conditions. The reaction was indeed performed at 100, 120, and 140 °C, leaving unaltered the other experimental parameters (i.e., concentration, molar ratio 1:3, reaction solvent) while reducing the reaction time. After 90 min of residence time at 140 °C the desired compound **7** was obtained in 6% yield (Supporting Information (SI), Table S1, entry 3). Longer residence time did not result in any significant improvement in terms of isolated yield (SI, Table S1, entry 4). Changing the molar ratio (2:1) between CBG and formaldehyde, afforded compound **7** in 12% yield, comparable with the result obtained in batch but in a significantly reduced reaction time (48 h vs 90 min). The possibility of using acidic or basic catalysts was also investigated. Treatment with *p*-toluensulfonic acid (*p*-TSA) led to extensive degradation of the starting material and the formation of a complex mixture of products. Substantial degradation was observed also in the presence of NaOH or DBU. Based on the results obtained by Astarloa-Aierbe et al.^21^ on phenolic resols resins, further attempts were carried out using triethylamine (TEA) (Scheme 2). Gratifyingly, employing 5 equiv of TEA while maintaining all the other experimental parameters unaltered, the yield of compound **7** was increased from 12% to 20% (SI, Table S1, entry 8). The best experimental conditions identified for the production of cannabizetol (**7**) were then applied for the continuous synthesis of cannabitwinol (**6**) that was obtained in 9% yield after only 90 min.

**Scheme 2:**
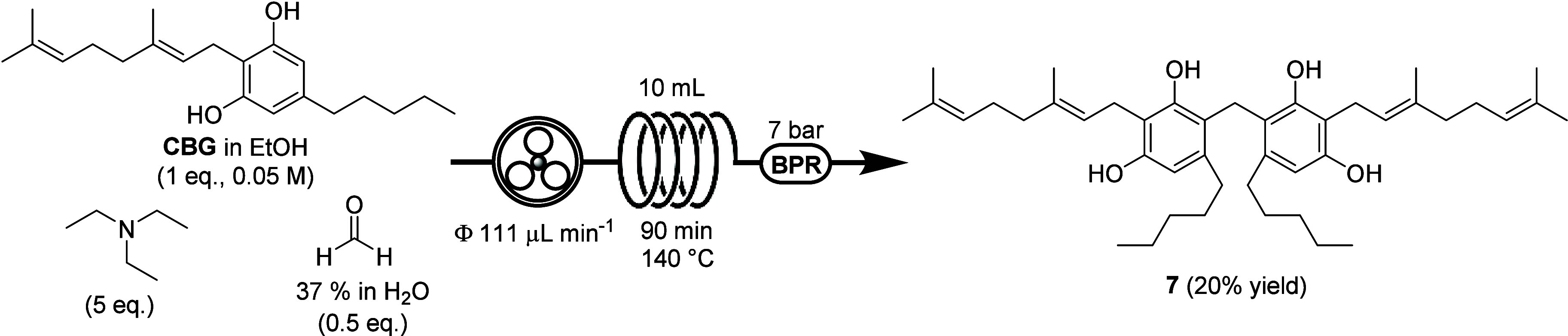
Best Experimental Condition Identified for the Continuous Synthesis Cannabizetol (**7**)

### Structural Characterization of Cannabizetol

All proton and carbon resonances of cannabizetol (**7**) were assigned (Table 1).

**Table 1:**
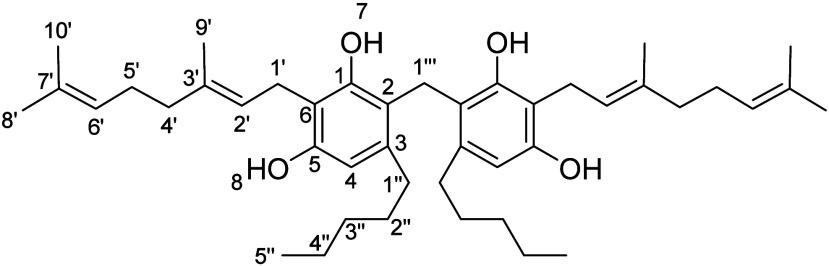
^1^H (400 MHz) and ^13^C (100 MHz) NMR Data of Cannabizetol (**7**) at 25 °C in MeOH-*d*_4_

position	δC, type	δH, mult (*J* in Hz)
1	154.6, C	
2	141.5, C	
3	118.4, C	
4	110.0, CH	6.22, s
5	154.7, C	
6	114.5, C	
7 (−OH)		
8 (−OH)		
1′	23.5, CH_2_	3.31–3.29^a^, m
2′	124.7, CH	5.21, t (*J* = 6.8 Hz)
3′	135.5, C	
4′	41.0, CH_2_	2.00–1.91, m
5′	27.8, CH_2_	2.10–2.01, m
6′	125.5, CH	5.07, t (*J* = 7.0 Hz)
7′	132.1, C	
8′	17.8, CH_3_	1.56, s
9′	16.3, CH_3_	1.74, s
10′	25.9, CH_3_	1.62, s
1″	34.6, CH_2_	2.48, t (*J* = 7.8 Hz)
2″	32.3, CH_2_	1.36–1.19, m
3″	33.2, CH_2_	1.36–1.19, m
4″	23.7, CH_2_	1.36–1.19, m
5″	14.4, CH_3_	0.86, t (*J* = 6.8 Hz)
1‴	23.7, CH_2_	3.89, s

a Overlapped to the solvent signal

The ^1^H NMR (400 MHz) spectra of cannabizetol (**7**), acquired at 25 °C in MeOH-*d*_4_ show a significant similarity to the NMR data of CBG in the same solvent (SI, Figure S6). The symmetrical nature of the molecule was immediately evident, and the characteristic diagnostic signals of CBG were preserved, showing an aromatic singlet at δ_H_ 6.22, two alkenyl triplets at δ_H_ 5.21 and δ_H_ 5.07, and three methyl singlets at δ_H_ 1.74, δ_H_ 1.62, and δ_H_ 1.56. The appearance of a singlet at δ_H_ 3.89, assigned to the methylene bridge of the dimer, suggested the successful formation of the desired compound, in analogy with those of compounds **6** (δ_H_ 3.85 in MeOH-*d*_4_) and **5** (δ_H_ 3.95 in CDCl_3_). Further confirmation of the correct assignment of the peak at δ_H_ 3.89 to the CH_2_–1‴ protons came from its COSY interactions with the aromatic proton CH-4 and its HMBC interactions with the quaternary aromatic carbons C-1, C-2, and C-3 (SI, Figure S7–S11). Copies of the 1D and 2D spectra are included in the Supporting Information. Further confirmation was provided by HRMS-ESI analysis, which displayed the molecular ion [M + H]^+^ at *m*/*z* 645.4895 (calcd 645.4883 for C_43_H_65_O_4_^+^).

### Isolation of Cannabizetol from *Cannabis sativa* Extracts

The synthesis and chemical characterization of dimer **7** proved to be crucial, as it enabled us to successfully identify and isolate it from *Cannabis sativa* chemotype IV extracts, which is characterized by a prevalence of CBG (>0.3%) and CBD (<0.5%).^22–24^ Starting from the aerial part of cannabis raw material, we extracted a crude phytocomplex with suitable organic solvents. Several purification steps allowed to obtain pure cannabigerol and mother liquors very rich in minor cannabinoids. Preparative HPLC treatment of mother liquors (C18 column, MeOH/H_2_O 80:20 v/v as mobile phase, run for 60 min, flow rate of 8 mL/min) allowed to obtain a fraction containing compound **7** with a purity higher than 90%; then the final purification by flash chromatography (from 1:1 to 6:4 CH_2_Cl_2_/*n*-hexane v/v as eluent) led to pure cannabizetol **7**. NMR spectra, ESI-MS and HPLC analysis (SI, Figure S25) demonstrated the exact match with the synthetic dimer **7**: in this way we confirmed the existence of a new cannabinoid in the plant extracts, the third methylene-bridged dimer.

### Inhibition of the NF-κB Driven Transcription and IL-8 Release in HaCaT Cells

Having pure dimers **6** and **7** available, and according with the results of our previous work,^18^ we decided to test the two compounds for their activity in inhibiting the NF-κB pathway (driven transcription) and IL-8 release in human keratinocyte HaCaT cells, which are considered the most useful model for investigating inflammatory-related diseases of the skin.^25^ Moreover, we previously demonstrated that CBD and CBG inhibit the NF-κB pathway in HaCaT cells^19,26^ thus making interesting a comparison with the corresponding dimers. Interleukin-8 (IL-8) plays a key role in various skin conditions, such as atopic dermatitis, psoriasis, and infections.^27,28^ Furthermore, IL-8 is a well-known chemokine regulated by NF-κB, crucial for the recruitment of neutrophils during inflammatory processes, including dermatitis.^29^ Therefore, the compounds were tested for cytotoxicity and then we evaluated their ability to inhibit the chemokine release, which is induced by tumor necrosis factor-alpha (TNFα) in HaCaT cells. Both compounds **6** and **7** did not show any cytotoxicity when tested in HaCaT cells at concentrations ranging between 0.5 and 20 μM by MTT assay (SI, Figure S1). Moreover, dimers showed a concentration dependent inhibition of TNFα-induced IL-8 release, with low IC_50_ values (6.39 μM and 1.46 μM for **6** and **7**, respectively). In particular, **7** showed higher activity than **6**, with significant inhibition of IL-8 release already at 1 μM (−30%) and complete inhibition at 5 μM (100%) (Figure 3A,B).^16^

**Figure 3:**
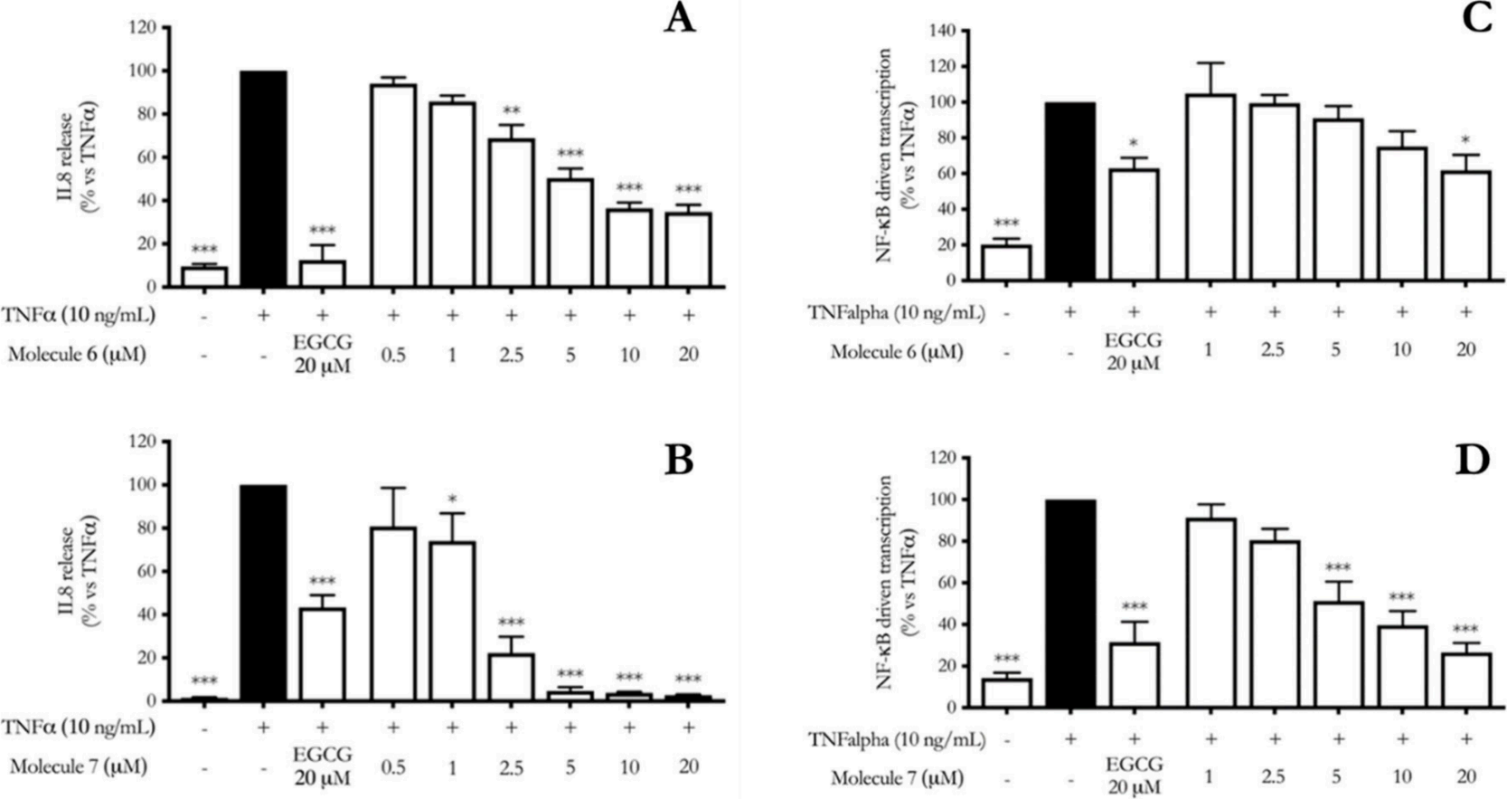
Effect of cannabinoids **6** (A) and **7** (B), on IL-8 release in HaCaT cells cotreated with the molecules and TNFα for 6 h. EGCG (20 μM) was used as reference compound. Data are reported as percentage in comparison to the stimulated control (mean ± SEM), which was arbitrarily assigned 100% value. * *p* < 0.05, *** *p* < 0.001 versus TNFα; on right, effect of cannabinoids **6** (C) and **7** (D), on NF-κB driven transcription in HaCaT cells cotreated with pure compounds and TNFα for 6 h. EGCG (20 μM) was used as reference compound. Data are reported as percentage in comparison to the stimulated control (mean ± SEM), which was arbitrarily assigned 100% value. * *p* < 0.05, *** *p* < 0.001 versus TNFα.

Since IL-8 is a well-known chemokine dependent by NF-κB activation, and we previously demonstrated CBD and CBG activities on this parameter, both **6** and **7** were tested to assess their ability to impair the NF-κB pathway in human keratinocytes. Both dimers showed inhibition of TNFα-induced NF-κB driven transcription. The effect of **6** was significantly lower than **7**, with a statistically significant effect only at 20 μM. Of note, **7** exerted a concentration dependent inhibition of the NF-κB driven transcription, with low IC_50_ (4.95 μM) (Figure 3C,D) whereas IC_50_ of **6** was significantly higher (19.8 μM). The interesting effect of **7** as inhibitor of both NF-κB driven transcription and IL-8 release, which are parameters strictly related to skin inflammatory conditions, prompted us to more in depth investigate the mechanisms underlying this effect. Then, an array was carried out in comparison with the dimer precursor, CBG. The array showed 25 pro-inflammatory genes upregulated by TNFα 10 ng/mL, including chemokines (i.e., CXCL8, CXCL10), interleukins (i.e., IL-1β) and cytokines (i.e., TNFα and LTB) (SI, Figure S2). CBG was able to downregulate two genes (CCL5 and CCL2) in a statistically significant way (SI, Figure S3A). Surprisingly, the effect of compound **7** was significantly higher than the corresponding monomer, exerting downregulation of 17 proinflammatory genes, in statistically significant way, deeply involved in skin inflammation, including CXCL8, CCL20 and CXCL1 (SI, Figure S3B).

## Conclusions

In this work, we expanded the library of known dimeric cannabinoids by identifying the novel compound cannabizetol (**7**) in *Cannabis sativa* extracts. This was only possible through its previous synthesis and full characterization. Subsequently, continuous synthesis of dimers **6** and **7** was investigated, finding the best conditions capable of achieving yields similar to batch synthesis, but in significantly shorter times. This study then evaluated the inhibition of the NF-κB driven transcription and IL-8 release in HaCaT cells of dimers **6** and **7**, demonstrating their significant biological activity, particularly of compound **7**. Moreover, dimer **7** was able to downregulate a variety of pro-inflammatory genes upregulated by TNFα whereas CBG failed to significantly modulate most of them. These results suggest that among the many still unknown cannabinoids there are also methylene-bridged dimers of other cannabinoids, including dimers composed of two different cannabinoids, with potential biological activities of great interest. As shown in this work, the synthesis of analytical standards could be useful in facilitating the identification of these compounds in cannabis extracts.

## Experimental Section

### General Experimental Procedures

^1^H NMR, ^13^C NMR, COSY, HSQC, and HMBC spectra were recorded at 298 K on a Brüker Avance Spectrometer (400 MHz), using commercially available deuterated solvents (CDCl_3_, MeOD). Chemical shifts are reported in parts per million (δ ppm), relative to internal TMS (^1^H, δ = 0.00 ppm), CDCl_3_ (^1^H, δ = 7.26 ppm; ^13^C, δ = 77.0 ppm) and MeOD (^1^H, δ = 3.31 ppm; ^13^C, δ = 49.0 ppm). Coupling constants (*J*) are given in hertz (Hz) and are quoted to the nearest 0.5 Hz. Peak multiplicities are described as follows: s, singlet; bs, broad singlet; d, doublet; t, triplet; m, multiplet; br, broad. HRMS spectra were recorded using an electrospray ionization (ESI) technique on FT-ICR APEXII (Bruker Daltonics, Bremen, Germany). HPLC was performed on Agilent 1100 Series system using a RP column ZORBAX SB C8 (3.5 μm × 4.6 mm × 150 mm) and with a gradient of H_2_O/MeOH ranging from 80% MeOH up to 100% MeOH in 30 min (flux of 1.2 mL/min, sample injection of 5 μL, sample concentration of 2 mg/mL in MeOH). The UV spectra was recorded at 254 and 220 nm with DAD detection. Continuous flow reactions were performed using E-series easy-Scholar system (Vaportec), equipped with PTFE coil reactor (10 mL). The temperature sensor sits on the wall of the reactors. Pressure was controlled by using back-pressure regulators.

### General Synthesis Procedures

Oven-dried glassware was used to perform chemical reactions, and dry solvents under a nitrogen (or argon if specified) atmosphere were employed. Solvents were purchased from Sigma-Aldrich and used as such. Chemical reagents were purchased from Merck (Milan, Italy), Fluorochem (Hadfield, United Kingdom), TCI (Zwijndrecht, Belgium) or BLDPharm (Hamburg, Germany) and used without further purification. CBD and CBG was extracted from *Cannabis sativa* as reported in a previously published protocol.^30^ Reaction monitoring by thin layer chromatography (TLC) on silica gel (Merck precoated 60 F_254_ plates), using UV light at 254 nm as a direct detection method or by staining by molybdic reagent. Purification of intermediates and final products was carried out by flash chromatography using high purity grade silica gel (Merck grade, pore size 60 Å, 230–400 mesh particle size, Sigma-Aldrich, Milan, Italy) as a stationary phase.

### General Procedure for Formaldehyde-Promoted Dimerization

Formaldehyde 37% w/w in H_2_O (6.0 equiv) was added dropwise to a solution of CBD (1.0 equiv) or CBG (1.0 equiv) in EtOH. The reaction mixture was left stirring at reflux (78 °C) for 48 h, then concentrated under reduced pressure. The crude was purified by flash column chromatography (from 1:1 to 6:4 CH_2_Cl_2_/*n*-hexane v/v as eluent).

#### Cannabitwinol (**6**)

Formaldehyde 37% w/w in H_2_O (143 μL, 1.92 mmol, 6.0 equiv), CBD (200.0 mg, 0.64 mmol, 1.0 equiv), EtOH (12.8 mL), 16% yield (32 mg), brownish solid. ^1^H NMR (400 MHz, MeOD): δ 6.14 (s, 2H), 5.42 (s, 2H), 4.40 (d, *J* = 10.7 Hz, 4H), 4.05–3.92 (m, 2H), 3.85 (s, 2H), 2.68 (s, 2H), 2.42 (t, *J* = 7.3 Hz, 4H), 2.30–2.16 (m, 2H), 2.12–1.98 (m, 2H), 1.86–1.69 (m, 10H), 1.62 (s, 6H), 1.39–1.18 (m, 12H), 0.87 (t, *J* = 7.5 Hz, 6H). ^13^C NMR (100 MHz, MeOD): δ 155.1, 149.3, 142.1, 138.2, 126.6, 119.0, 115.7, 111.3, 109.7, 47.1, 37.7, 34.3, 33.3, 32.0, 31.6, 30.2, 23.9, 23.7, 23.0, 19.5, 14.5. HRMS (ESI+), *m*/*z* [M + H]^+^: calculated for C_43_H_61_O_4_^+^ 641.4570; found 641.4574.

#### Cannabizetol (**7**)

Formaldehyde 37% w/w in H_2_O (141 μL, 1.89 mmol, 6.0 equiv), CBG (200.0 mg, 0.63 mmol, 1.0 equiv), EtOH (12.6 mL), 13% yield (24 mg), brownish solid. ^1^H NMR (400 MHz, MeOD): δ 6.22 (s, 2H), 5.21 (t, *J* = 6.8 Hz, 2H), 5.07 (t, *J* = 7.0 Hz, 2H), 3.90 (s, 2H), 3.29 (s, 4H), 2.48 (t, *J* = 7.8 Hz, 4H), 2.10–2.01 (m, 4H), 2.00–1.91 (m, 4H), 1.74 (s, 6H), 1.62 (s, 6H), 1.56 (s, 6H), 1.36–1.19 (m, 12H), 0.86 (t, *J* = 6.8 Hz, 6H). ^13^C NMR (101 MHz, MeOD): δ 154.7, 154.6, 141.5, 135.5, 132.1, 125.5, 124.7, 118.4, 114.5, 110.0, 40.9, 34.6, 33.2, 32.3, 27.7, 25.9, 23.7, 23.5, 17.8, 16.3, 14.4. HRMS (ESI+), *m*/*z* [M + H]^+^: calculated for C_43_H_65_O_4_^+^ 645.4883; found 645.4881.

### Synthesis of (*E*)-2-(3,7-Dimethylocta-2,6-dien-1-yl)-1,3-dimethoxy-5-pentylbenzene (**8**)

Cs_2_CO_3_ (821 mg, 2.52 mmol, 4.0 equiv) and iodomethane (392 μL, 6.30 mmol, 10.0 equiv) were added to a solution of CBG (200.0 mg, 0.63 mmol, 1.0 equiv) in MeCN (2.1 mL) at rt. The reaction mixture was left stirring at reflux (80 °C) for 5 h, then H_2_O (10 mL) was added. The aqueous phase was extracted with CH_2_Cl_2_ (3 × 20 mL), then the collected organic phases were dried over Na_2_SO_4_ and concentrated under reduced pressure. Compound **8** was obtained with 89% yield (192 mg) as a transparent oil after flash column chromatography purification (from 95:5 to 85:15 *n*-hexane/CH_2_Cl_2_ *v/v* as eluent). ^1^H NMR (400 MHz, CDCl_3_): δ 6.37 (s, 2H), 5.19 (t, *J* = 7.1 Hz, 1H), 5.07 (t, *J* = 7.0 Hz, 1H), 3.80 (s, 6H), 3.31 (d, *J* = 7.1 Hz, 2H), 2.56 (t, *J* = 7.9 Hz, 2H), 2.10–1.99 (m, 2H), 1.98–1.91 (m, 2H), 1.75 (s, 3H), 1.64 (s, 3H), 1.63–1.58 (m, 2H), 1.57 (s, 3H), 1.39–1.29 (m, 4H), 0.90 (t, *J* = 6.4 Hz, 3H). ^13^C NMR (101 MHz, CDCl_3_): δ 158.1, 141.9, 134.3, 131.1, 124.7, 123.3, 115.8, 104.2, 55.8, 40.0, 36.7, 31.8, 31.4, 26.9, 25.8, 22.7, 22.2, 17.8, 16.1, 14.2. HRMS (ESI+), *m*/*z* [M + H]^+^: calculated for C_23_H_37_O_2_^+^ 345.2788; found 345.2782.

### Synthesis of (*E*)-3-(3,7-Dimethylocta-2,6-dien-1-yl)-2,4-dihydroxy-6-pentylbenzaldehyde (**9**)

POCl_3_ (740 μL, 7.90 mmol, 2.5 equiv) was added dropwise to a solution of CBG (1.00 g, 3.16 mmol, 1.0 equiv) in DMF (6.3 mL) cooled at 0 °C. The reaction mixture was left stirring at rt for 21 h, then cooled at 0 °C. A saturated NaHCO_3_ solution (30 mL) was added, then the aqueous phase was extracted with EtOAc (3 × 50 mL). The collected organic phases were dried over Na_2_SO_4_ and concentrated under reduced pressure. Compound **9** was obtained with 50% yield (549 mg) as a white solid after flash column chromatography purification (from 6:4 to 4:6 *n*-hexane/CH_2_Cl_2_ *v/v* as eluent). ^1^H NMR (400 MHz, CDCl_3_): δ 12.80 (s, 1H), 10.06 (s, 1H), 6.22 (s, 1H), 6.20 (s, 1H), 5.27 (t, *J* = 7.4 Hz, 1H), 5.08–5.00 (m, 1H), 3.41 (d, *J* = 7.4 Hz, 2H), 2.79 (t, *J* = 7.6 Hz, 2H), 2.16–2.03 (m, 4H), 1.81 (s, 3H), 1.67 (s, 3H), 1.66–1.60 (m, 2H), 1.59 (s, 3H), 1.39–1.30 (m, 4H), 0.89 (t, *J* = 7.0 Hz). ^13^C NMR (101 MHz, CDCl_3_): δ 193.3, 164.1, 163.1, 147.8, 140.3, 132.6, 124.1, 121.4, 112.8, 111.9, 110.4, 40.2, 32.9, 32.1, 32.1, 26.8, 26.1, 22.9, 21.7, 18.2, 16.7, 14.4. HRMS (ESI+), *m*/*z* [M + H]^+^: calculated for C_22_H_33_O_3_^+^ 345.2424; found 345.2428.

### Synthesis of (*E*)-3-(3,7-Dimethylocta-2,6-dien-1-yl)-2,4-dimethoxy-6-pentylbenzaldehyde (**10**)

Cs_2_CO_3_ (951 mg, 2.92 mmol, 4.0 equiv) and iodomethane (455 μL, 7.30 mmol, 10.0 equiv) were added to a solution of intermediate **9** (250 mg, 0.73 mmol, 1.0 equiv) in MeCN/CH_2_Cl_2_ (7.3 mL) at rt. The reaction mixture was left stirring at reflux (80 °C) for 2 h, then H_2_O (15 mL) was added. The aqueous phase was extracted with CH_2_Cl_2_ (3 × 30 mL), then the collected organic phases were dried over Na_2_SO_4_ and concentrated under reduced pressure. Compound **10** was obtained in quantitative yield (266 mg) as a transparent oil without any further purification. ^1^H NMR (400 MHz, CDCl_3_): δ 10.37 (s, 1H), 6.52 (s, 1H), 5.19–5.12 (m, 1H), 5.09–5.01 (m, 1H), 3.88 (s, 3H), 3.81 (s, 3H), 3.33 (d, *J* = 6.8 Hz, 2H), 3.00–2.91 (m, 2H), 2.10–2.02 (m, 2H), 2.01–1.94 (m, 2H), 1.77 (s, 3H), 1.63 (s, 3H), 1.57 (s, 5H), 1.43–1.31 (m, 4H), 0.91 (t, *J* = 6.9 Hz, 3H). ^13^C NMR (101 MHz, CDCl_3_): δ 191.6, 164.5, 162.9, 147.4, 135.7, 131.8, 124.7, 123.0, 122.0, 121.0, 109.5, 64.5, 56.2, 40.2, 34.9, 32.5, 31.7, 27.1, 26.1, 23.0, 22.8, 18.1, 16.6, 14.5. HRMS (ESI+), *m*/*z* [M + H]^+^: calculated for C_24_H_37_O_3_^+^ 373.2737; found 373.2741.

### Synthesis of (*E*)-(3-(3,7-Dimethylocta-2,6-dien-1-yl)-2,4-dimethoxy-6-pentylphenyl)­methanol (**11**)

NaBH_4_ (272 mg, 7.30 mmol, 10.0 equiv) was added to a solution of intermediate **10** (266 mg, 0.73 mmol, 1.0 equiv) in MeOH (3.6 mL) cooled at 0 °C. The reaction mixture was left stirring at 0 °C for 2 h, then H_2_O (10 mL) was added. The aqueous phase was extracted with CH_2_Cl_2_ (3 × 20 mL), then the collected organic phases were dried over Na_2_SO_4_ and concentrated under reduced pressure. Compound **11** was obtained in quantitative yield (265 mg) as a transparent oil without any further purification. ^1^H NMR (400 MHz, CDCl_3_): δ 6.52 (s, 1H), 5.19 (t, *J* = 6.5 Hz, 1H), 5.06 (t, *J* = 6.6 Hz, 1H), 4.68 (s, 2H), 3.81 (s, 3H), 3.79 (s, 3H), 3.33 (d, *J* = 6.5 Hz, 2H), 2.73–2.62 (m, 2H), 2.09–2.01 (m, 2H), 2.00–1.93 (m, 2H), 1.76 (s, 3H), 1.64 (s, 3H), 1.57 (s, 5H), 1.43–1.30 (m, 4H), 0.91 (t, *J* = 6.9 Hz, 3H). ^13^C NMR (101 MHz, MeOH-*d*_4_): δ 159.4, 159.4, 143.6, 135.1, 132.0, 125.4, 125.0, 124.9, 121.8, 108.8, 63.1, 56.5, 56.0, 40.8, 34.0, 33.2, 32.8, 27.6, 25.9, 23.8, 23.6, 17.7, 16.3, 14.4. HRMS (ESI+), *m*/*z* [M + H]^+^: calculated for C_24_H_39_O_3_^+^ 375.2894; found 375.2888.

### Synthesis of Bis­(3-((*E*)-3,7-dimethylocta-2,6-dien-1-yl)-2,4-dimethoxy-6-pentylphenyl)­methane (**12**)

A solution of compound **11** (49 mg, 0.13 mmol, 1.0 equiv) in CH_2_Cl_2_ (4.0 mL) was added dropwise to a solution of compound **8** (79 mg, 0.23 mmol, 1.75 equiv) and *p*-TSA (27 mg, 0.14 mmol, 1.1 equiv) in CH_2_Cl_2_ (0.3 mL) cooled at 0 °C. The reaction mixture was left stirring at 0 °C for 2 h, then washed with saturated NaHCO_3_ solution (10 mL) and H_2_O (5 mL). The organic phase was dried over Na_2_SO_4_ and concentrated under reduced pressure. Compound **12** was obtained with 46% yield (39 mg) as a yellowish oil after gravimetric column chromatography purification (from 95:5 to 85:15 *n*-hexane/CH_2_Cl_2_ *v/v* as eluent). ^1^H NMR (400 MHz, CDCl_3_): δ 6.41 (s, 2H), 5.23 (t, *J* = 6.7 Hz, 2H), 5.12–5.03 (m, 2H), 4.08 (s, 2H), 3.77 (s, 6H), 3.60 (s, 6H), 3.36 (d, *J* = 6.7 Hz, 4H), 2.40 (t, *J* = 8.3 Hz, 4H), 2.12–2.03 (m, 4H), 2.03–1.95 (m, 4H), 1.78 (s, 6H), 1.65 (s, 6H), 1.59 (s, 6H), 1.22–0.97 (m, 12H), 0.81 (t, *J* = 6.9 Hz, 6H). ^13^C NMR (101 MHz, CDCl_3_): δ 157.8, 157.0, 142.0, 134.6, 131.5, 126.2, 125.0, 124.6, 121.0, 108.6, 61.9, 56.0, 40.3, 34.0, 32.5, 31.3, 27.2, 26.1, 23.8, 23.2, 23.1, 18.1, 16.6, 14.4. HRMS (ESI+), *m*/*z* [M + H]^+^: calculated for C_47_H_73_O_4_^+^ 701.5503; found 701.5499.

### Synthesis of Cannabizetol (**7**)

Under Ar atmosphere at rt, pentamethyldisiloxane (51 μL, 0.26 mmol, 4.4 equiv) was added to a solution of intermediate **12** (44 mg, 0.06 mmol, 1.0 equiv) in *n*-heptane (270 μL). After 5 min, BCF (0.6 mg, 0.0012 mmol, 0.02 equiv) was added and the reaction mixture was left stirring at rt for 14 h. A saturated NaCl solution (10 mL) was added, then the aqueous phase was extracted with EtOAc (3 × 20 mL). The collected organic phases were dried over Na_2_SO_4_ and concentrated under reduced pressure. The crude was dissolved under Ar atmosphere in THF (600 μL), then cooled at 0 °C. A 1 M solution of TBAF in dry THF (720 μL, 0.72 mmol TBAF, 12 equiv) was added dropwise, and the reaction mixture was left stirring at rt for 1 h. A saturated NH_4_Cl solution (10 mL) was added, then the aqueous phase was extracted with EtOAc (3 × 20 mL). The collected organic phases were dried over Na_2_SO_4_ and concentrated under reduced pressure. Cannabizetol **7** was obtained with 33% yield (10 mg) as a brownish solid after flash column chromatography purification (from 1:1 to 4:6 *n*-hexane/CH_2_Cl_2_ v/v as eluent). Spectroscopic data are consistent with those described before.

### Continuous Synthesis of Cannabitwinol (**6**) and Cannabizetol (**7**)

Stock solution was prepared solubilizing the monomer in EtOH (CBG or CBD, 1 equiv, 0.05M). Triethylamine (5 equiv) and then 0.5 equiv of formaldehyde 37% in H_2_O were added. The solution was pumped through a 10 mL coil thanks to a peristaltic pump at 111 μL min^–1^. The reactor was heated at 140 °C and pressurized at 7 bar (back pressure regulator BPR). The exiting flow was collected, the solvent removed under reduced pressure and the crude purified by flash column chromatography (from 1:1 to 6:4 CH_2_Cl_2_/*n*-hexane v/v as eluent).

### Isolation of Cannabizetol from *Cannabis sativa*

The aerial parts (stem, leaves, flowers) of *Cannabis sativa* chemotype IV (origin: Europe, stored at room temperature in polyethylene bags) (250 kg) were extracted with EtOH (v/v) to afford 15 kg of a crude phytocomplex. HPLC analysis was performed to identify compound **7** by comparison with the synthetic standard and to evaluate its concentration (HPLC area% of approximately 1–2%). The extract was then subjected to column chromatography on Sephadex LH-20 (column internal diameter 19 mm, stationary phase height 18 cm). Five grams of crude extract were dissolved in CH_2_Cl_2_ (25 mL), loaded onto the column, and purified. Ten chromatographic runs were carried out, and the dimer-enriched fractions were pooled to yield 15.5 g of enriched material containing approximately 5% of dimer **7** (HPLC area%). The entire enriched extract was further purified by preparative HPLC (Shimadzu LC20 AP instrument equipped with an SPD-40 V detector set at 228 nm, customized column 250 mm × 25 mm, 15 μm Lichrospher RP18 stationary phase; flow rate 8.0 mL/min; injection volume 500 μL; sample concentration ∼925 mg/mL; mobile phase MeOH/H_2_O 80:20; isocratic run, 60 min). This process afforded 1.2 g of a mixture containing compound **7** with 90% purity (HPLC area %). Successive flash chromatographic purifications (CH_2_Cl_2_/*n*-hexane gradient from 1:1 to 6:4) afforded 20 mg of pure **7**. NMR and MS analyses confirmed an exact match with synthetic dimer **7**. We can therefore assume that the initial 250 kg of plant material contains between 50 and 150 g of cannabizetol **7**, meaning that its presence in the extract is below 0.1%.

### Cell Line

HaCaT cells, spontaneously immortalized human keratinocyte line,^31^ were kindly provided by Cell Line Service GmbH (Eppelheim, Germany). Cells were grown in DMEM (Gibco, ThermoFisher Scientific, Waltham, Massachusetts, USA) supplemented with 10% heat-inactivated fetal bovine serum (Euroclone S.p.A., Milan, Italy), l-glutamine (2 mM; Gibco, ThermoFisher Scientific, Waltham, Massachusetts, USA), penicillin (100 U/mL), and streptomycin (100 mg/mL; Gibco, ThermoFisher Scientific, Waltham, Massachusetts, USA), at 37 °C in humidified atmosphere containing 5% CO_2_.

Every 4 days, at 80–90% of confluence, cells were detached from the 75 cm^2^ flasks (Euroclone S.p.A., Milan, Italy) using tripsin-EDTA 0.25% (Gibco, ThermoFisher Scientific, Waltham, Massachusetts, USA), counted, and replaced in a new flask, at the density of 1.5 × 10^6^ cells per flask, to allow the cell line growth. The remaining cells were seeded in 24-well plates (Euroclone S.p.A., Milan, Italy) for the biological tests.

### Cytotoxicity and Inhibition of the NF-κB Driven Transcription and IL-8 Release

Cell viability was assessed by light microscope, before and after treatment with compounds. Cytotoxicity of cannabinoids was evaluated by the 3,4,5-dimethylthiazol-2-yl-2,5-diphenyltetrazolium bromide method (MTT assay) (Merck, Darmstadt, Germany) at the end of treatment. This method evaluates cell viability by measuring the activity of mitochondrial succinate dehydrogenase. The medium was removed after 6 h treatment, and 200 μL of MTT solution was added until the development of a violet color typical of formazan formation. Then, the MTT solution was removed, and 200 μL of 2-propanol/DMSO 90:10 was added to each well for formazan extraction. The absorbance was read spectrophotometrically at 570 nm (Envision, PerkinElmer, USA). IL-8 secretion in the medium was quantified by an enzyme-linked immunosorbent assay (ELISA) Kit (Peprotech, London, UK), HaCaT cells were grown in 24-well plates (Euroclone S.p.A., Milan, Italy) (30000 cells/well) for 72 h; then, cells were cotreated with TNFα (10 ng/mL) and the individual compounds for 6 h. Corning 96-well EIA/RIA plates (Merck, Darmstadt, Germany) were coated with the antibody provided in the ELISA Kit and incubated overnight at room temperature to allow the binding between the antibody and the bottom of the wells. After blocking phase, the samples were transferred into wells at room temperature for 2 h. The IL-8 in the samples was detected by the use of a biotinylated antibody and of HRP-conjugated avidin (horseradish peroxidase). The colorimetric reaction between HRP enzyme and 2,2′-azino-bis­(acido 3-etilbenzotiazolina-6-solfonico) (ABTS) (Merck, Darmstadt, Germany) was read using a spectrophotometer at 405 nm, 0.1s (Victor X3, PerkinElmer, Walthman MA, USA). The quantification of IL-8 was performed through a standard curve supplied with the ELISA Kit (0–1000 pg/mL). Data were expressed considering 100% the absorbance related to the TNFα-induced IL-8 release. In order to evaluate the NF-κB driven transcription, HaCaT cells were grown in 24-well plates (Euroclone S.p.A., Milan, Italy) (30000 cells/well) for 72 h, then the cells were transiently transfected by lipofectamine 3000 (ThermoFisher Scientific, Waltham, Massachusetts, USA) with the reporter plasmid NF-κB-Luc. After 6 h treatment with the stimulus TNFα and the molecule, the luciferase was detected using Britelite Plus Kit (Revvity, Waltham, Massachusetts, USA).

Following a 6 h treatment, the anti-inflammatory activity of the molecules was evaluated on 84 inflammatory genes using an RT-PCR array (RT^2^ Profiler PCR Array Human Inflammatory Cytokines and Receptors, QIAGEN S.r.l., Hilden, Germany), as previously described.^32^ HaCaT cells were lysed using QIAZOL Lysis Reagent (QIAGEN S.r.l., Hilden, Germany), and the RNA isolation was performed using the miRNeasy Mini Kit purchased from QIAGEN (QIAGEN S.r.l., Hilden, Germany). RT^2^ First Strand Kit from QIAGEN (QIAGEN S.r.l., Milan, Italy) was used for cDNA synthesis and genomic DNA elimination in RNA samples. An aliquot of cDNA, corresponding to 400 ng of total RNA, was then mixed with the SYBR Green Master Mix RT2 reagent (QIAGEN S.r.l., Milan, Italy) and loaded into the 384-well array. The RT-PCR was conducted using the CFX384TM Real-Time PCR Detection System, which was coupled to a C1000TM Thermal Cycler (Bio-Rad Laboratories S.r.l., Segrate, Italy). The threshold cycle value for each gene (CT) was automatically provided by the management software CFX ManagerTM 2.1 (Bio-Rad Laboratories S.r.l., Segrate, Italy), depending on the amplification curves. The cycle threshold (Ct) cutoff was fixed at 33, and the housekeeping gene RPLP0 was used for data normalization. The data analysis web portal employs the delta Ct method to calculate fold change/regulation, whereby Fold Change (FC) is determined as FC = 2^–ΔΔ*Ct*^ to quantify alterations in gene expression between the treated and control groups. The analysis was carried out using the web portal at GeneGlobe (QIAGEN S.r.l., Hilden, Germany).

### Statistical Analysis

All data were expressed as the mean ± SEM of at least three independent experiments. Gene expression results were calculated using the ΔΔ*Ct* method and the *p* values were calculated based on a Student’s *t* test. ELISA assay and NF-κB driven transcription were analyzed by unpaired one-way analysis of variance (ANOVA), followed by Bonferroni post hoc test. Statistical analyses were performed using GraphPad Prism 9.0 software (GraphPad Software Inc., San Diego, CA, USA). Values of *p* < 0.05 were considered statistically significant.

## Supplementary Material



## Data Availability

The NMR data for compounds **6**–**12** has been deposited in the Natural Products Magnetic Resonance Database (NP-MRD; https://https://np-mrd.org/) and can be found at NP0351482 (cannabitwinol **6**), NP0351483 (cannabizetol **7**), NP0351484 (**8**), NP0351485 (**9**), NP0351486 (**10**), NP0351487 (**11**), and NP0351488 (**12**).

## Notes

Reaction conditions screening for continuous synthesis of cannabizetol; Cytotoxicity assay of cannabinoids **6** and **7** in HaCaT cells; effect of TNFα on gene expression of 84 genes related to the inflammatory process; Effect of cannabigerol and **7** on TNFα-induced gene expression of 84 genes related to the inflammatory process; NMR spectra of intermediates and final compounds; HPLC chromatograms of cannabitwinol (**6**) and cannabizetol (**7**) (PDF)
