# Supplementary material for: Cannabizetol, a Novel Cannabinoid: Chemical Synthesis, Anti-inflammatory Activity and Extraction from Cannabis sativa L
Source: J Nat Prod. 2025 Sep 25;88(10):2451–9. doi: 10.1021/acs.jnatprod.5c00826 (PMC12560071; doi:10.1021/acs.jnatprod.5c00826)
Supplement: Supplementary file 1 [file np5c00826_si_001.pdf]

SUPPORTING INFORMATION

Cannabizetol, a Novel Cannabinoid: Chemical Synthesis, Anti-inflammatory Activity and Extraction from *Cannabis sativa* L.

Luca Pozzi,[a] Andrea Gotti,[b] Marco Fumagalli,[c] Andrea Citarella,[a] Valerio Fasano,[a] Giuseppe Paladino,[d] Umberto Ciriello,[d] Salvatore Princiotto,[b] Francesca Annunziata,[b] Giulia Martinelli,[c] Enrico Sangiovanni,[c] Andrea Pinto,[b] Mario Dell'Agli,[c] Daniele Passarella.*[a]

[a] *Department of Chemistry, Università degli Studi di Milano, 20133 Milan, Italy.*
[b] *Department of Food, Environmental and Nutritional Sciences (DeFENS), Università degli Studi di Milano, 20133 Milan, Italy.*
[c] *Department of Pharmacological and Biomolecular Sciences "Rodolfo Paoletti", Università degli Studi di Milano, 20133 Milan, Italy.*
[d] *LINNEA SA, 6595 Riazzino (TI), Switzerland.*

Corresponding: daniele.passarella@unimi.it

Contents

Contents	Pages
**1. Continuous reaction conditions screening for cannabizetol**	S1
**2. Cytotoxicity tests**	S2
**3. Gene expression tests**	S3 – S4
**4. ^{1}H and APT NMR spectra of cannabitwinol (6)**	S5 – S6
**5. ^{1}H, APT and 2D NMR spectra of cannabizetol (7)**	S7 – S12
**6. ^{1}H and APT NMR spectra of intermediates 8, 9, 10, 11, 12**	S13 – S22
**7. HPLC traces of cannabitwinol (6) and cannabizetol (7)**	S23 – S26

1. Continuous reaction conditions screening for cannabizetol

Supplementary Table S1: Reaction conditions screening for continuous synthesis of cannabizetol (**7**).

Entry	T (°C)	P (bar)	Res. time (min)	Molar ratio $CBG:CH_2O$	Catalyst Molar Ratio CBG:Cat.	Yield (%)
**1**	100	5	90	1:3	-	no product
**2**	120	5	90	1:3	-	traces
**3**	140	7	90	1:3	-	6
**4**	140	7	180	1:3	-	6
**5**	140	7	90	1:100	-	traces
**6**	140	7	90	1:0.5	-	12
**7**	140	7	90	1:0.5	*p*TSA (1:0.1)	degradation
**8**	140	7	90	1:0.5	TEA (1:5)	20
**9**	140	7	90	1:0.5	TEA (1:0.5)	12
**10**	140	7	90	1:0.5	TEA (1:1)	12
**11**	140	7	90	1:0.5	TEA (1:10)	12
**12**	140	7	60	1:0.5	TEA (1:5)	9
**13**	140	7	30	1:0.5	TEA (1:5)	3

2. Cytotoxicity tests

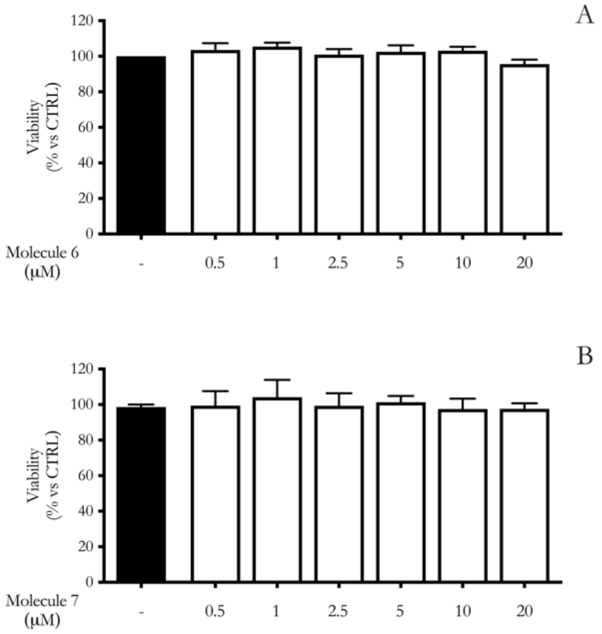

Supplementary Figure S1. Cytotoxicity assay (MTT test) of cannabinoids **6** (A) and **7** (B), in HaCaT cells treated with concentrations ranging from 0.5 to 20 μM. Data are reported as percentage in comparison to the control (mean ± SEM), which was arbitrarily assigned 100% value.

3. Gene expression tests

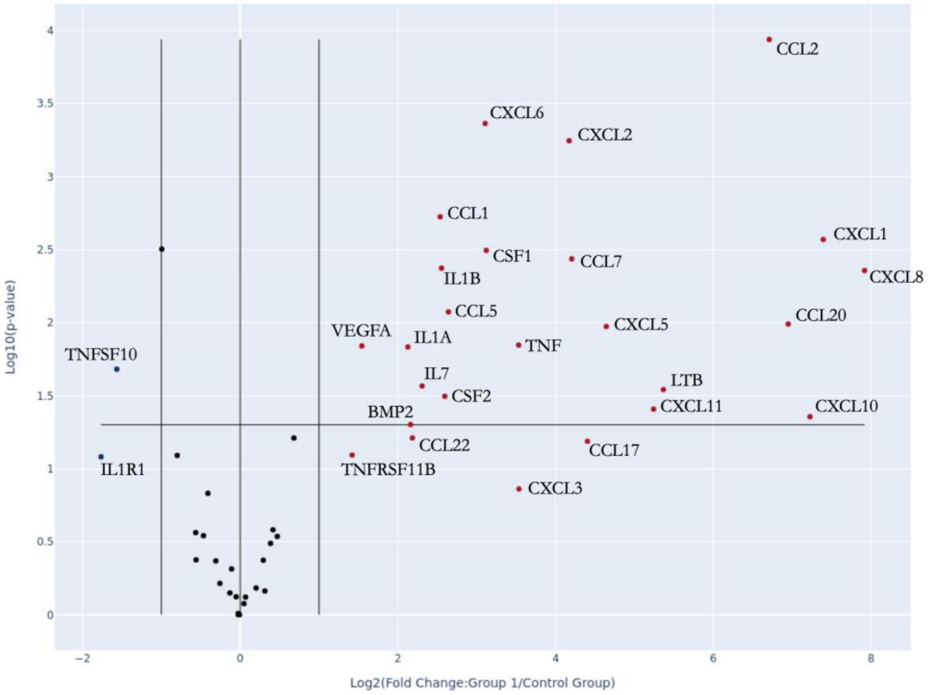

Supplementary Figure S2. Effect of TNFα (10 ng/mL) on gene expression of 84 genes related to the inflammatory process. The Volcano Plot identifies significant gene expression changes by plotting the $\log_2$ of the fold changes in gene expression on the x-axis versus their statistical significance on the y-axis (p-value threshold: 0.05). The center vertical line indicates unchanged gene expression, while the two outer vertical lines indicate the selected fold regulation threshold (fold regulation threshold: 2).

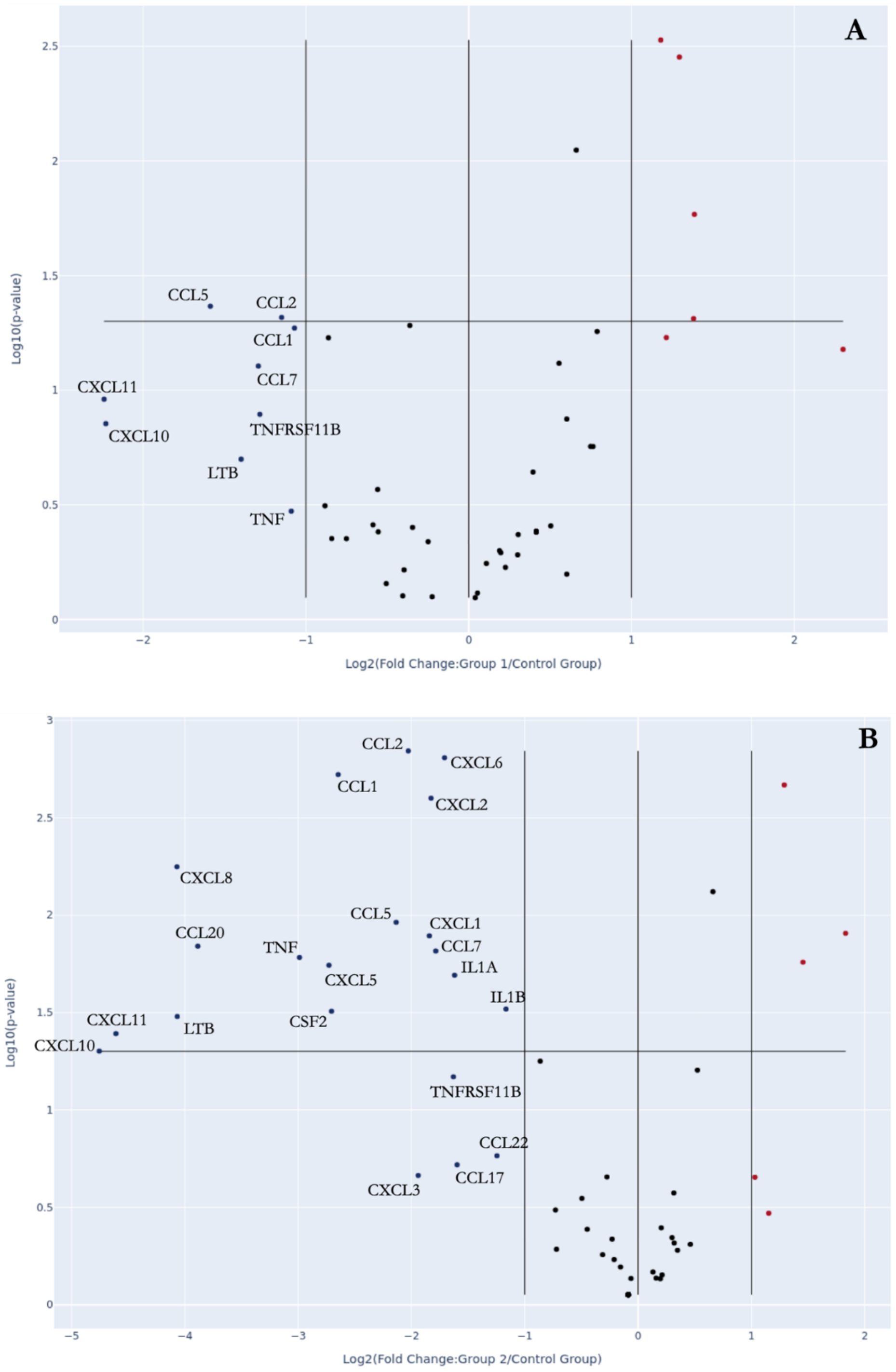

Supplementary Figure S3. Effect of cannabigerol (A) and compound **7** (B) at concentration of 5 µM on TNFα-induced gene expression of 84 genes related to the inflammatory process. The Volcano Plot identifies significant gene expression changes by plotting the $\log_2$ of the fold changes in gene expression on the x-axis versus their statistical significance on the y-axis (p-value threshold: 0.05). The center vertical line indicates unchanged gene expression, while the two outer vertical lines indicate the selected fold regulation threshold (fold regulation threshold: 2).

4. ^{1}H and APT NMR spectra of cannabitwinol (6)

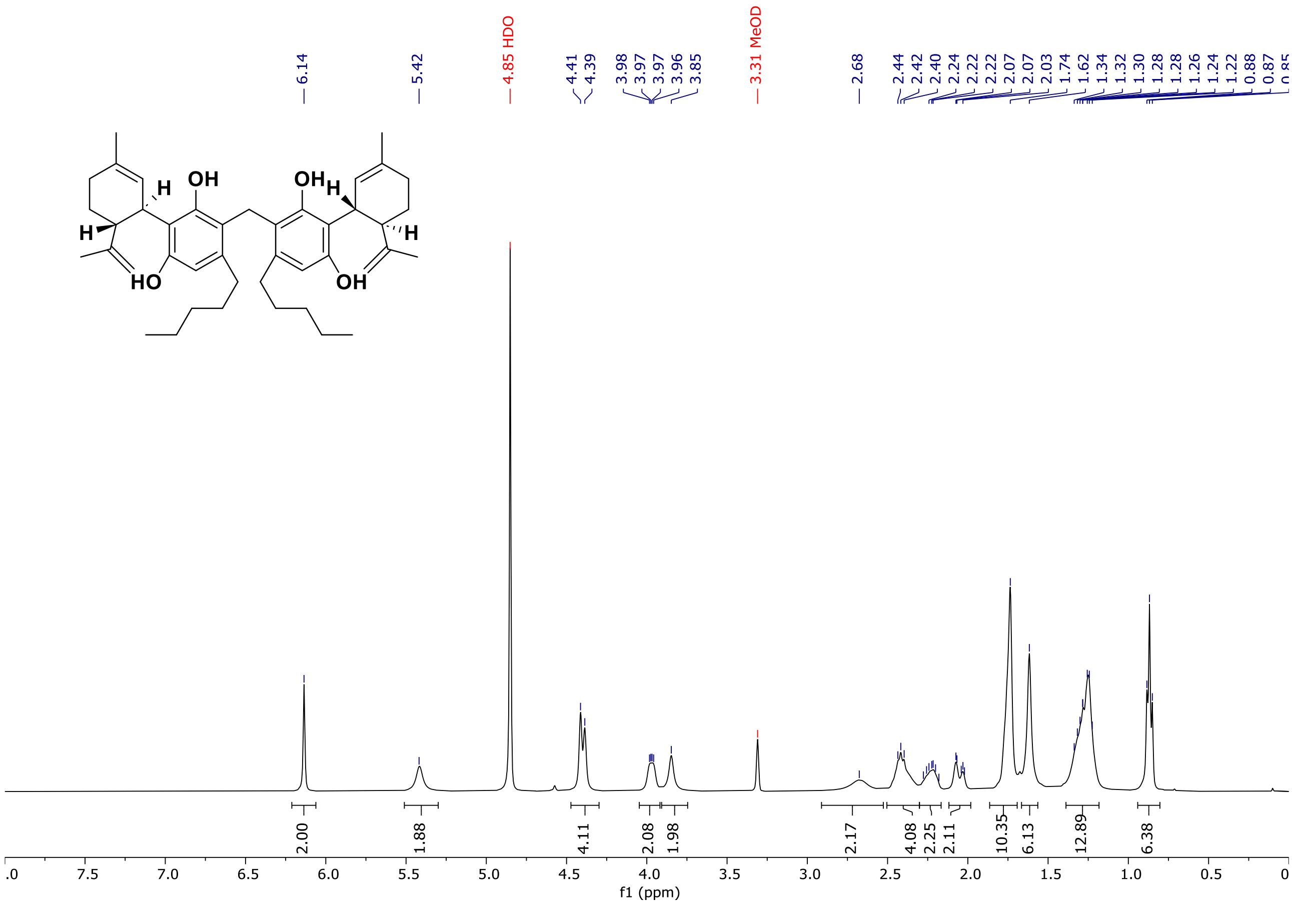

Supplementary Figure S4. ^{1}H-NMR spectra (400 MHz, CD_3OD) of **6.**

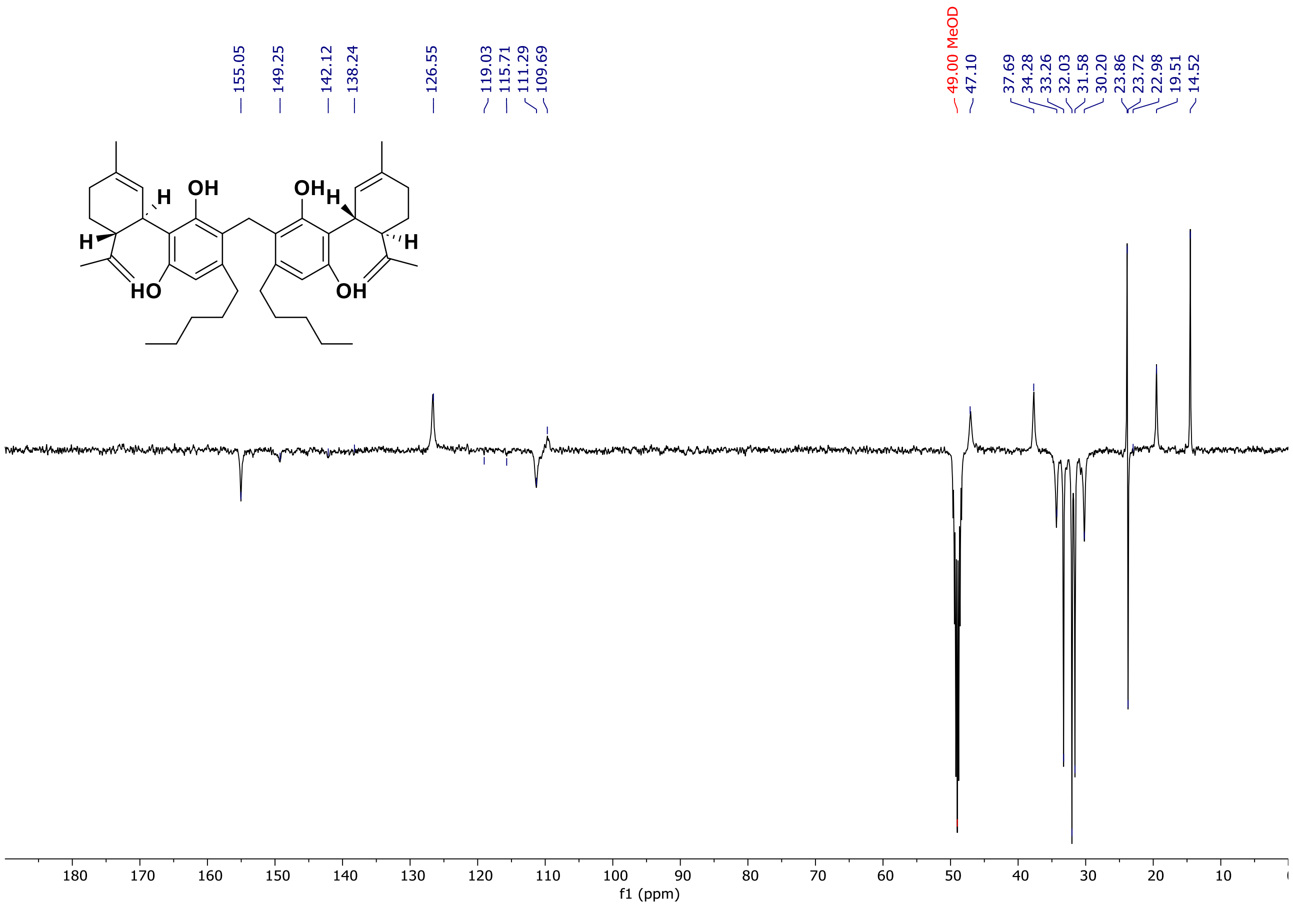

Supplementary Figure S5. APT NMR spectra (101 MHz, CD_3OD) of **6.**

5. ^{1}H, APT and 2D NMR spectra of cannabizetol (7)

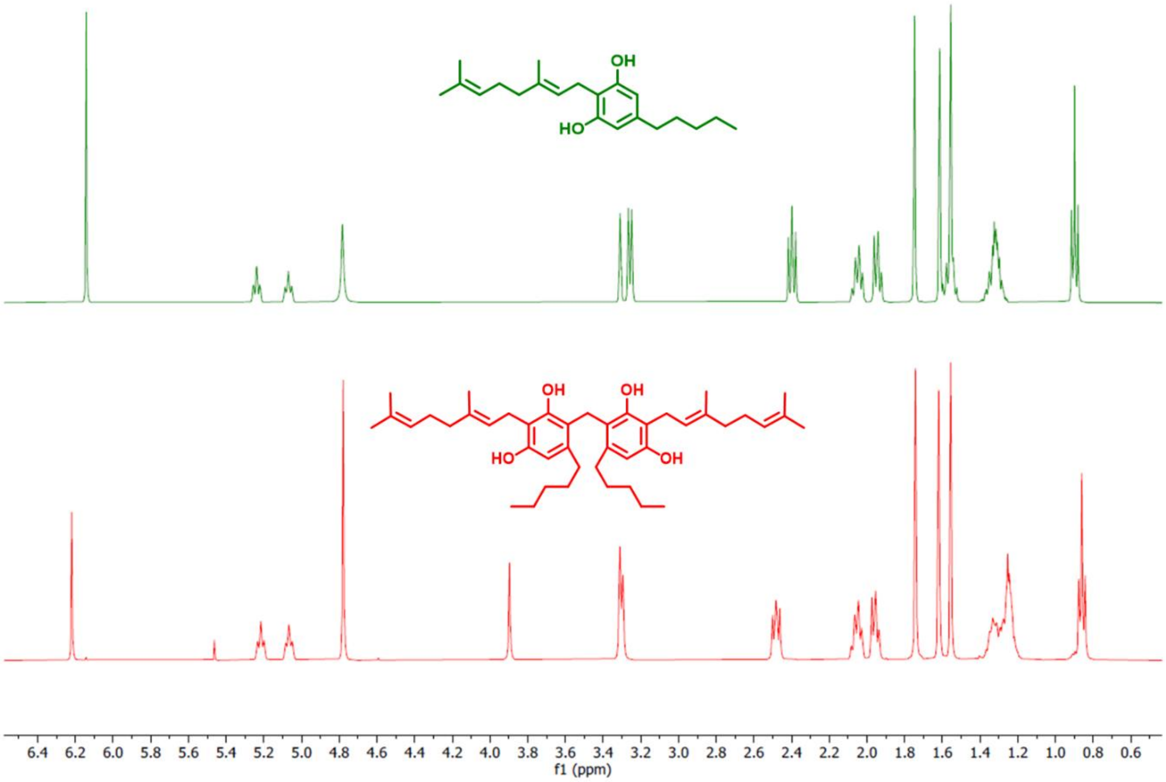

Supplementary Figure S6. Comparison of the ^{1}H NMR (400 MHz) spectra of CBG (on top, in green) and cannabizetol (on bottom, in red) in MeOH-d_4 at 25 °C.

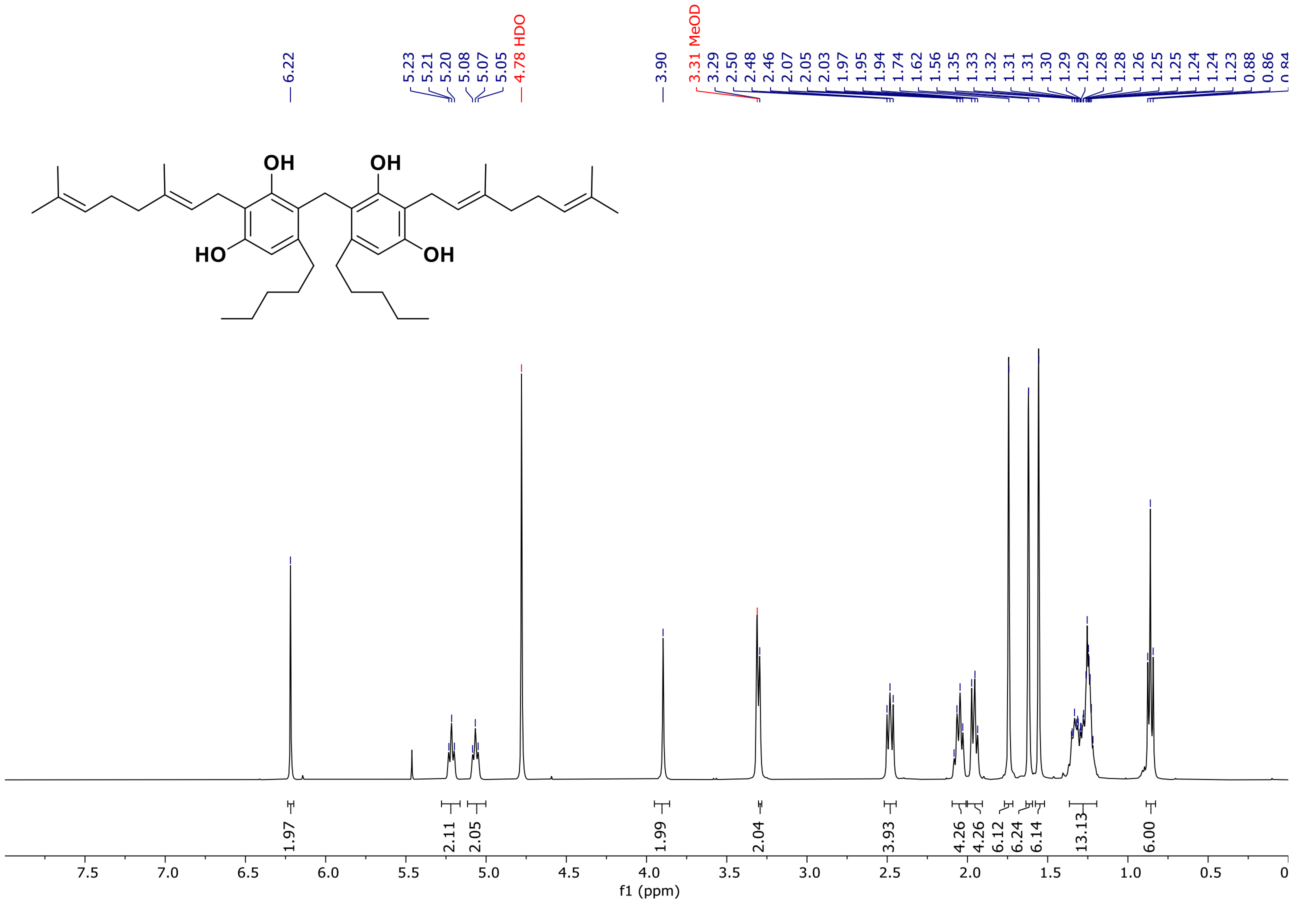

Supplementary Figure S7. ^{1}H-NMR spectra (400 MHz, MeOH-d_4) of **7.**

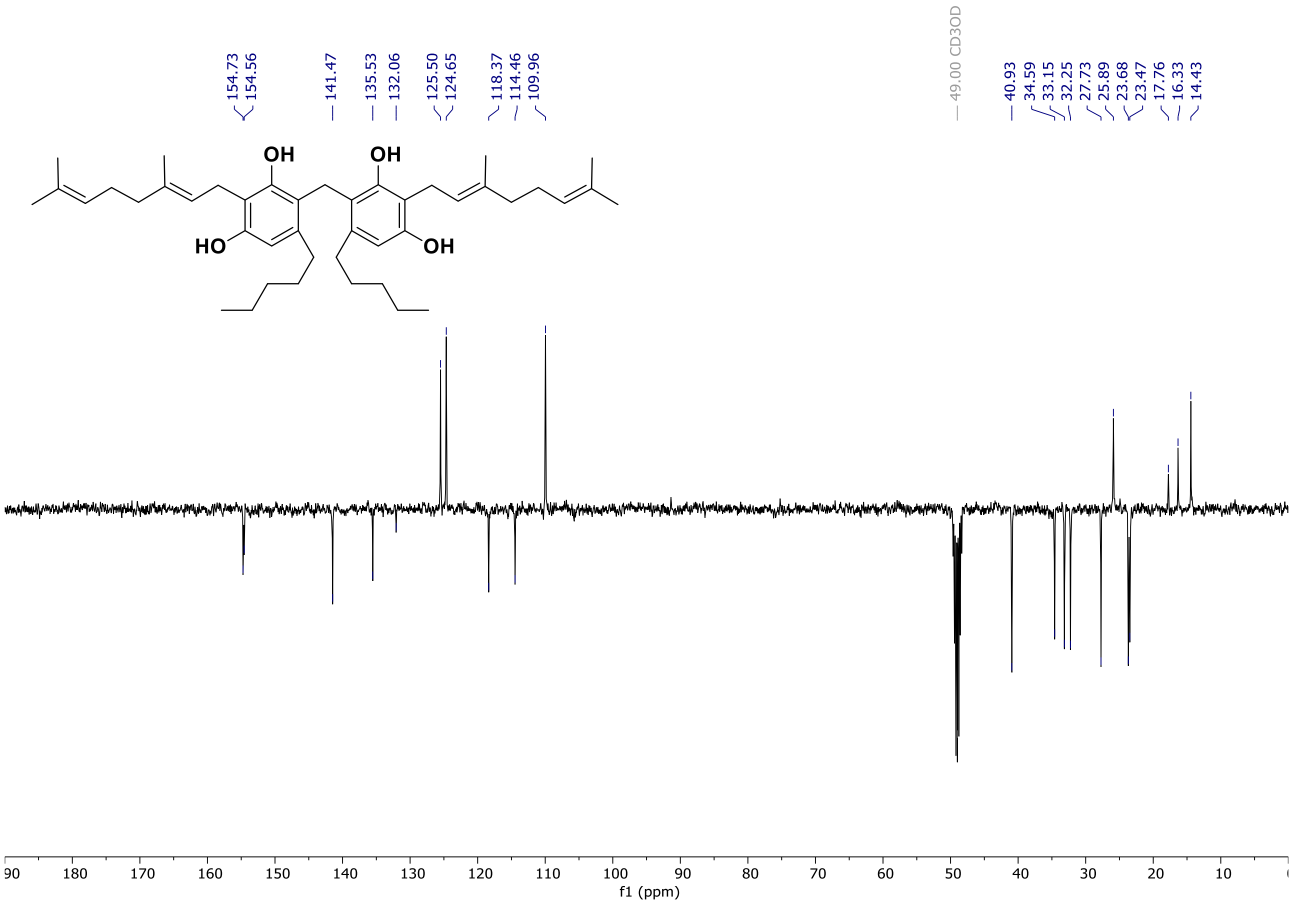

Supplementary Figure S8. APT NMR spectra (101 MHz, MeOH-d_4) of **7.**

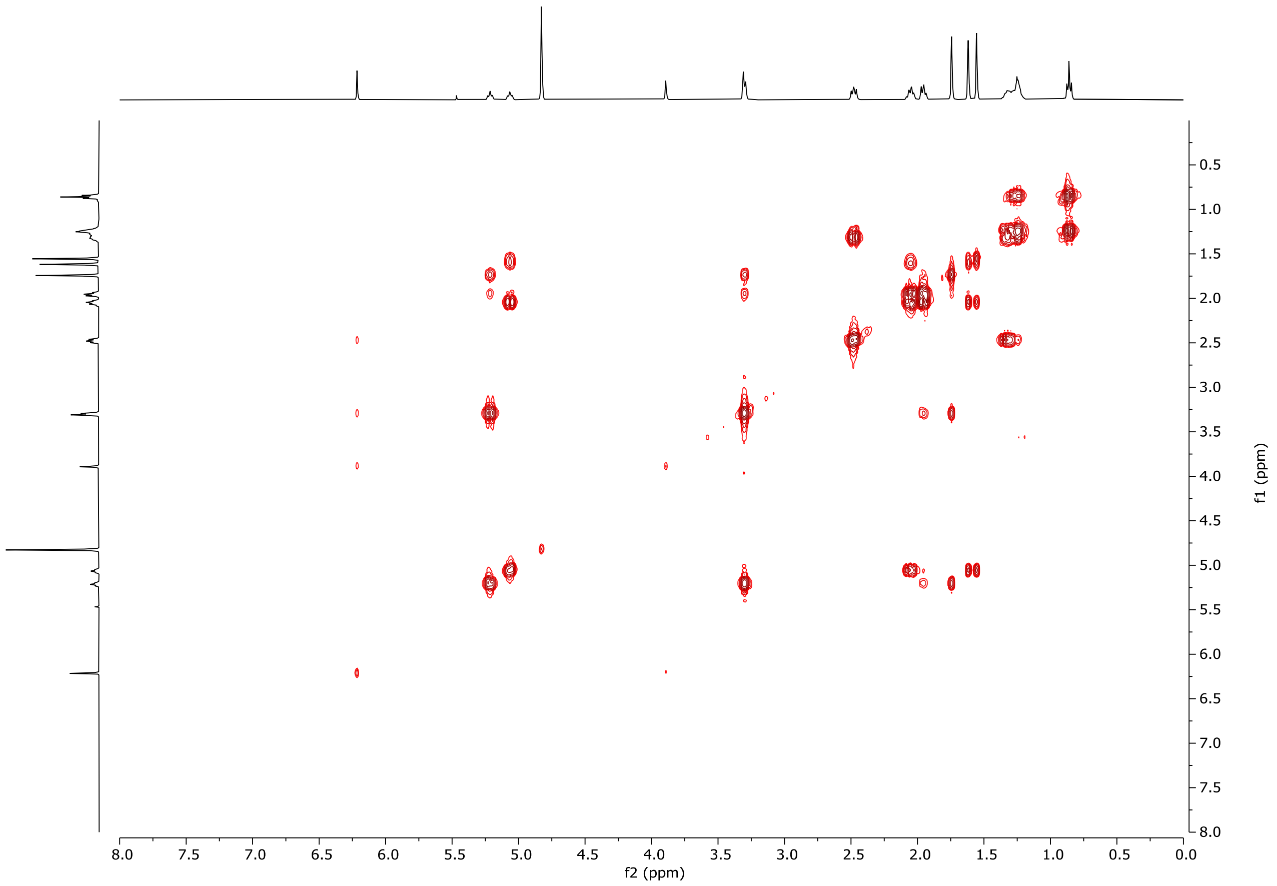

Supplementary Figure S9. 2D NMR COSY spectra of compound **7** in MeOH-d_4.

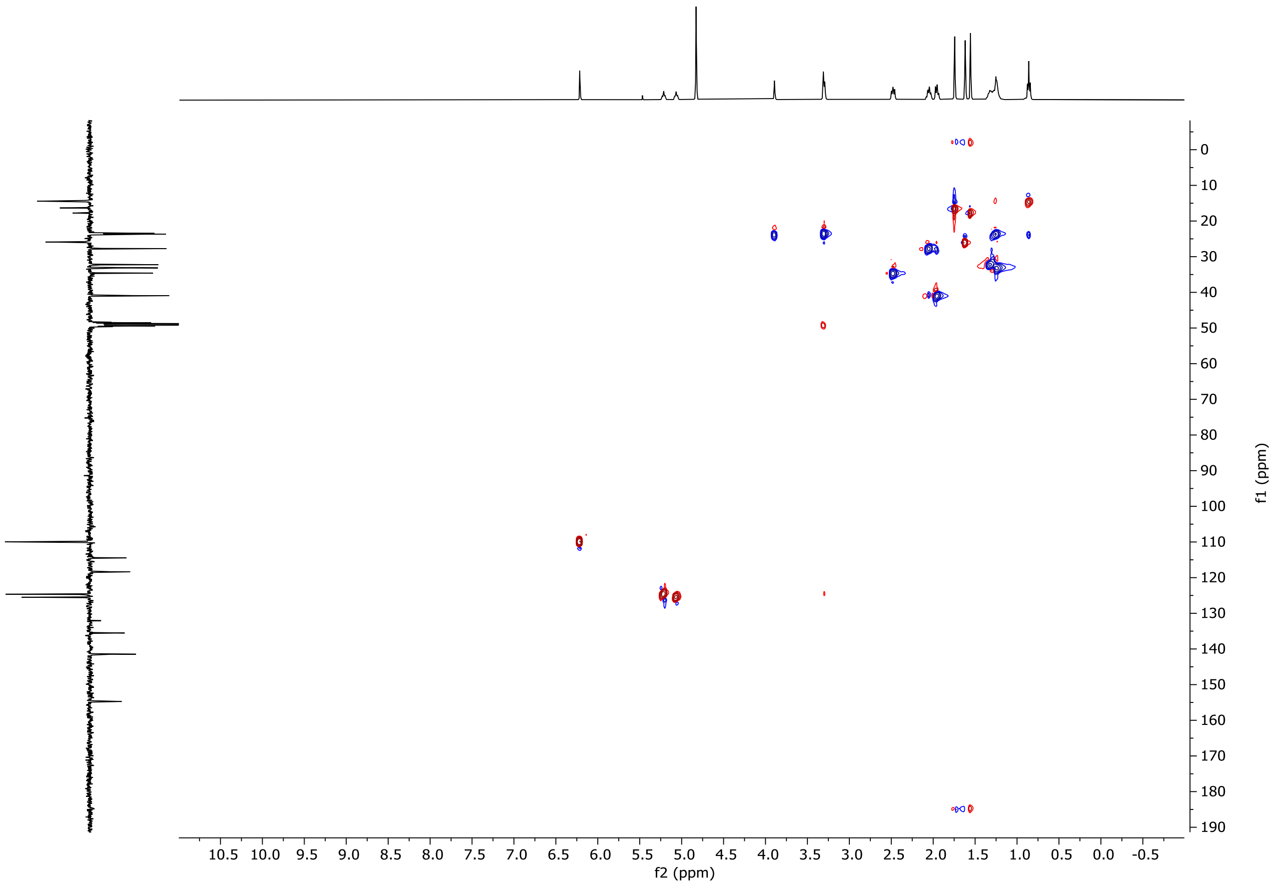

Supplementary Figure S10. 2D NMR HSQC spectra of compound **7** in MeOH-d_4.

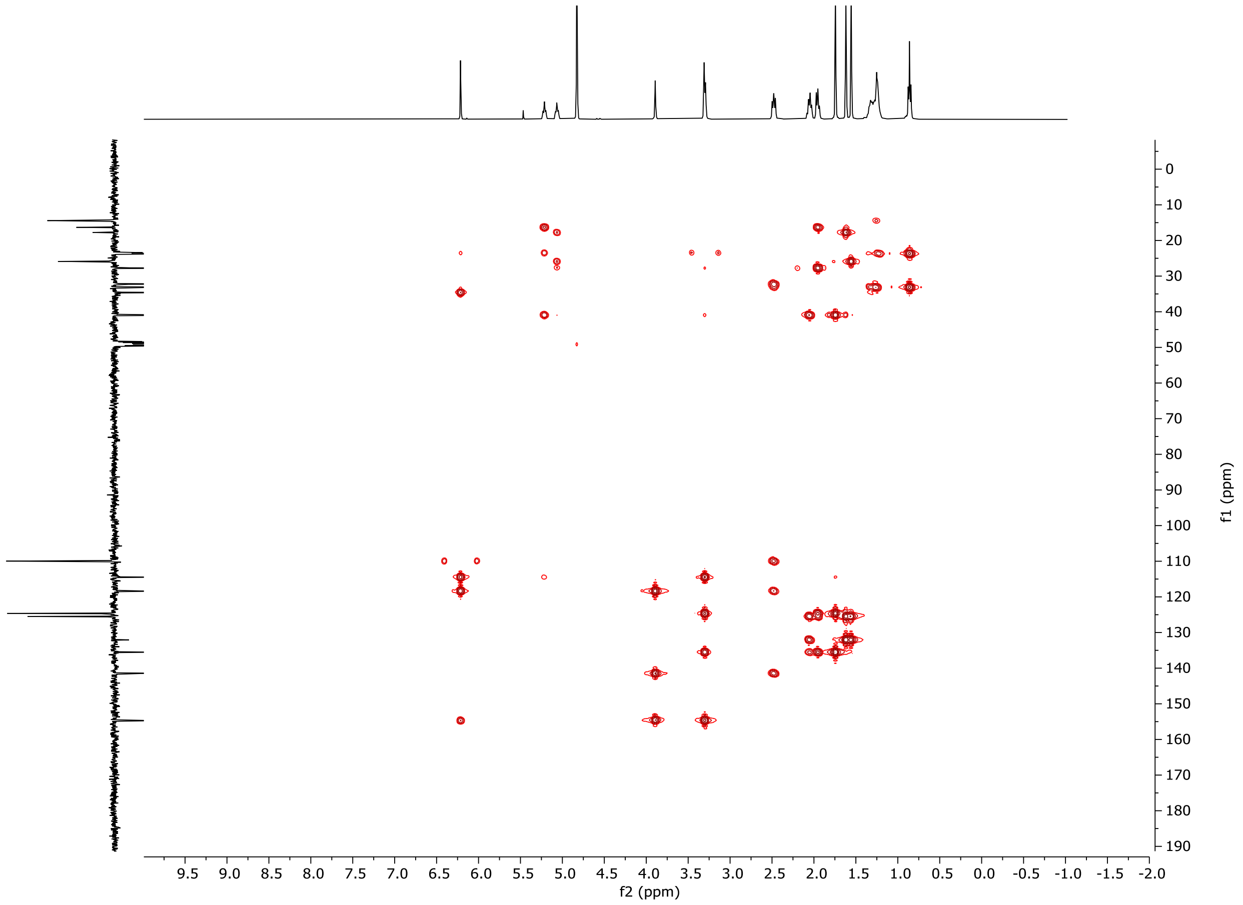

Supplementary Figure S11. 2D NMR HMBC spectra of compound **7** in MeOH-d_4.

6. ^{1}H and APT NMR spectra of intermediates 8, 9, 10, 11, 12

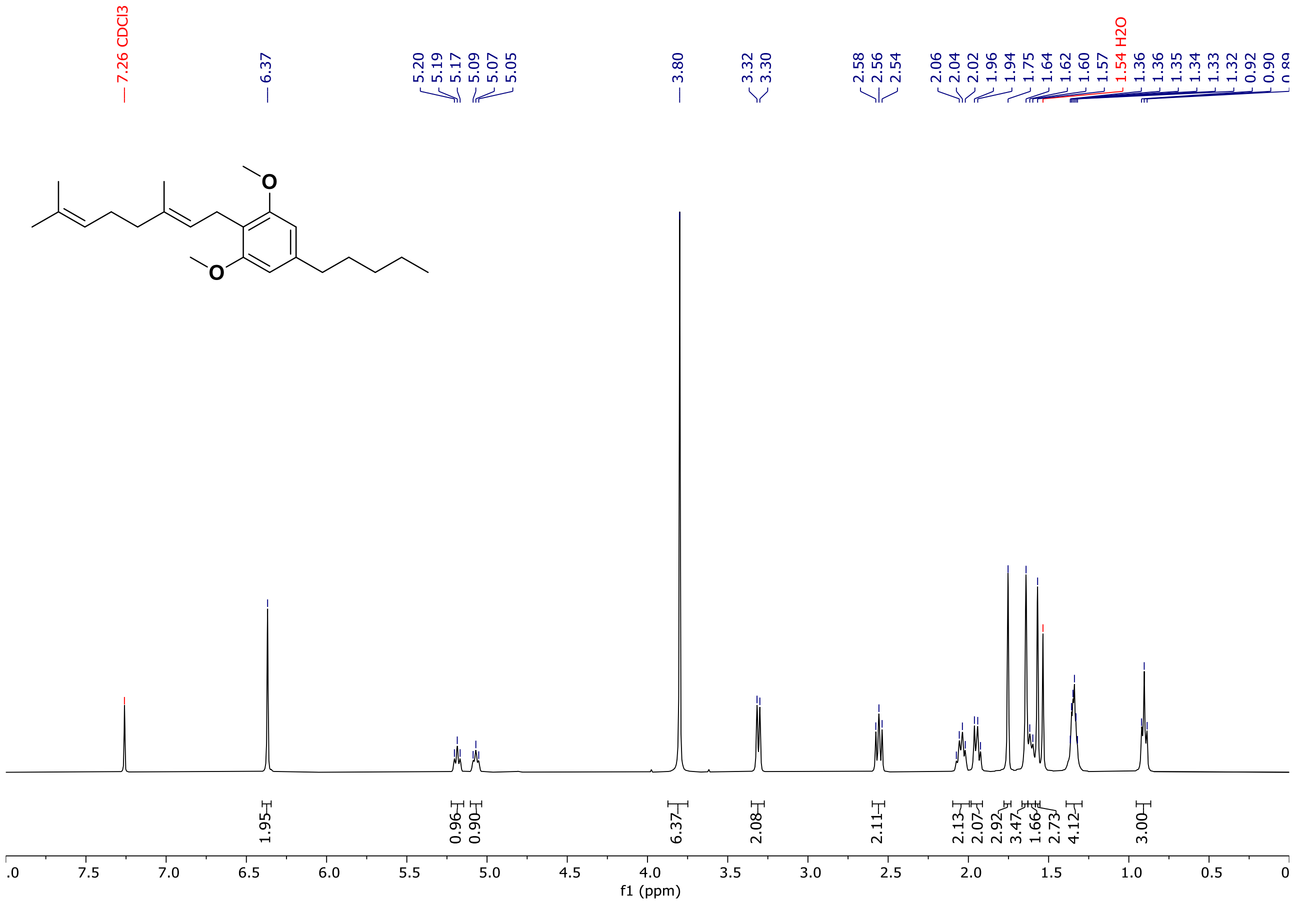

Supplementary Figure 12. ^{1}H-NMR spectra (400 MHz, $CDCl_3$) of **8.**

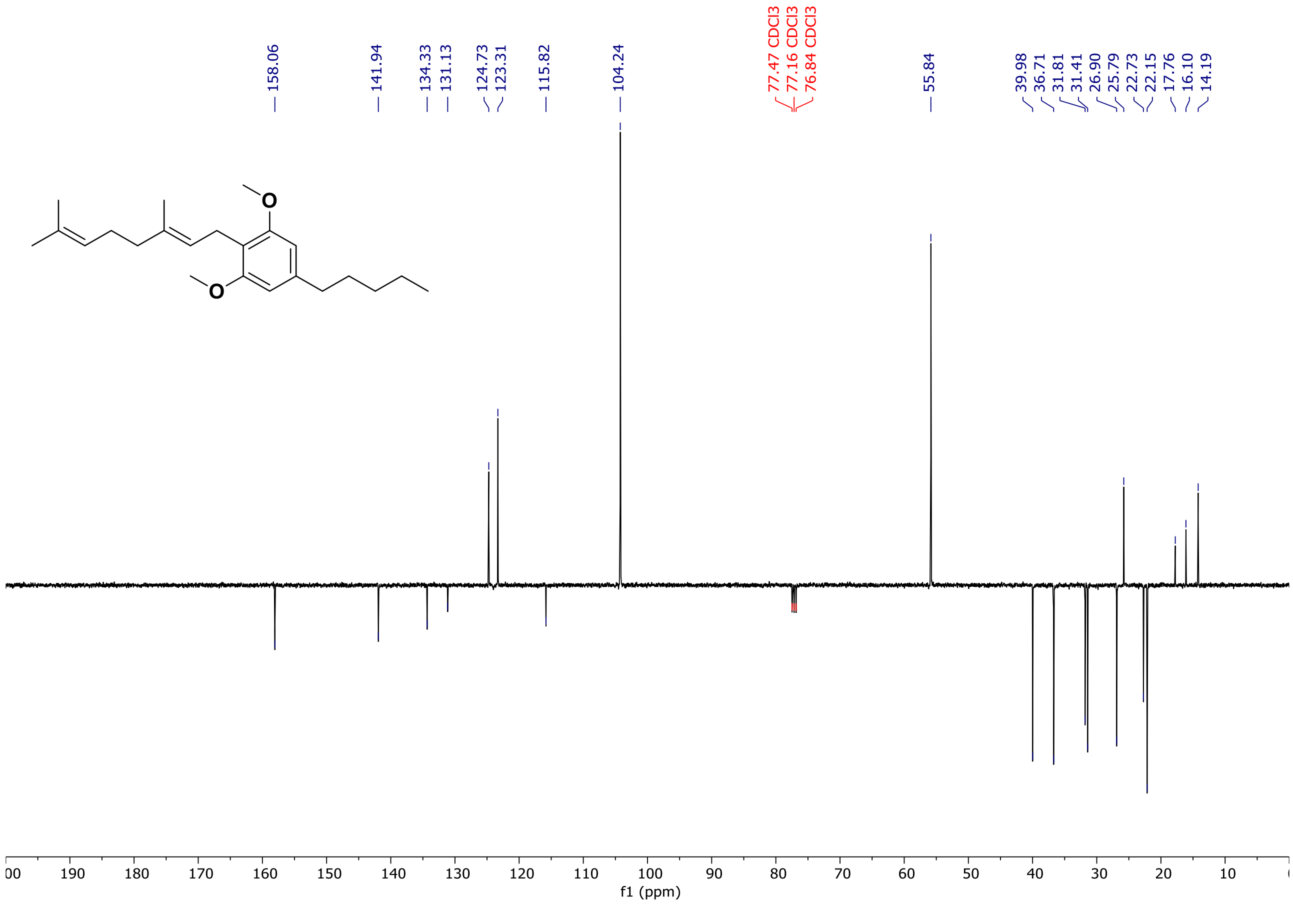

Supplementary Figure S13. APT NMR spectra (101 MHz, $CDCl_3$) of **8.**

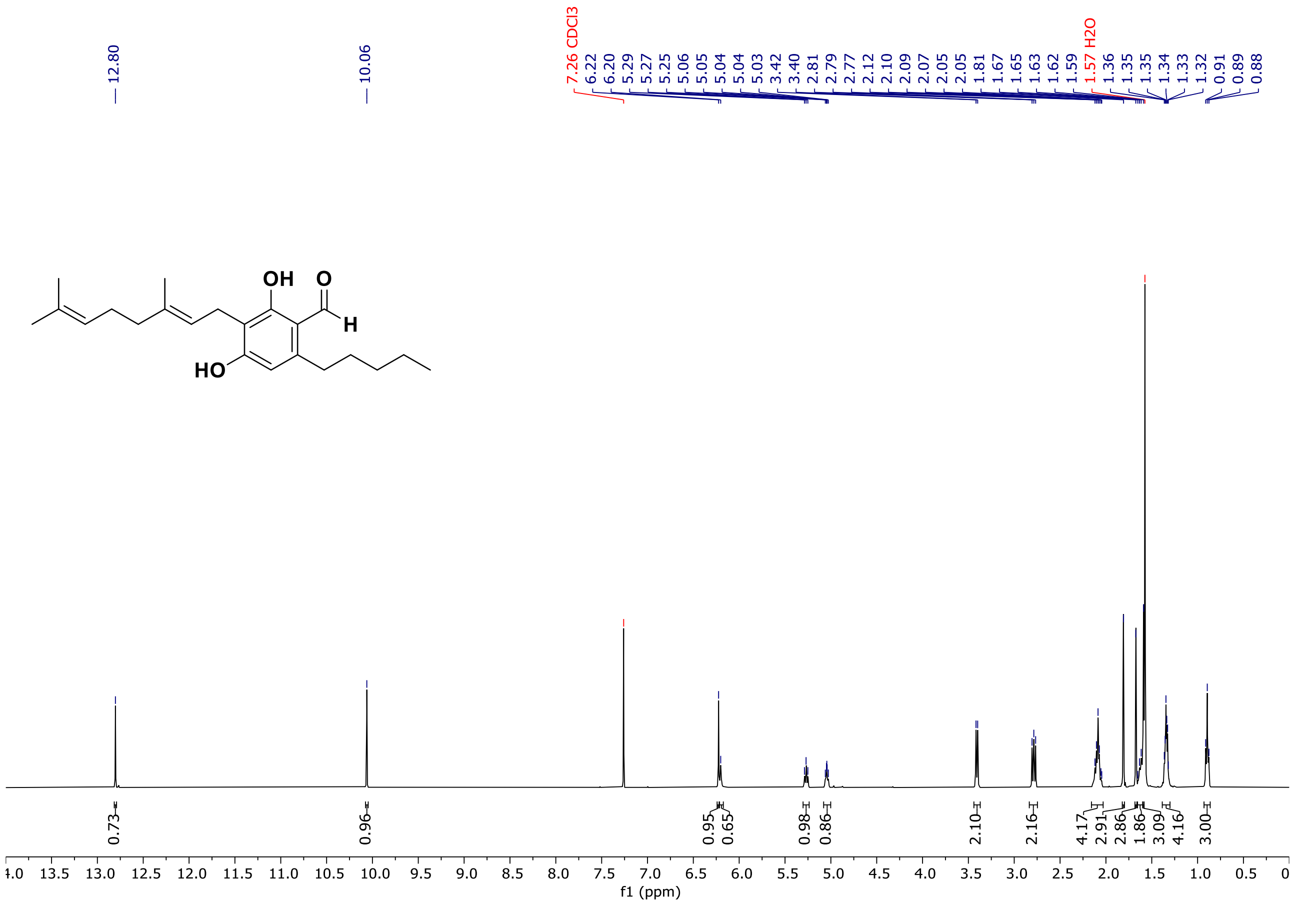

Supplementary Figure S14. ^{1}H-NMR spectra (400 MHz, $CDCl_3$) of **9.**

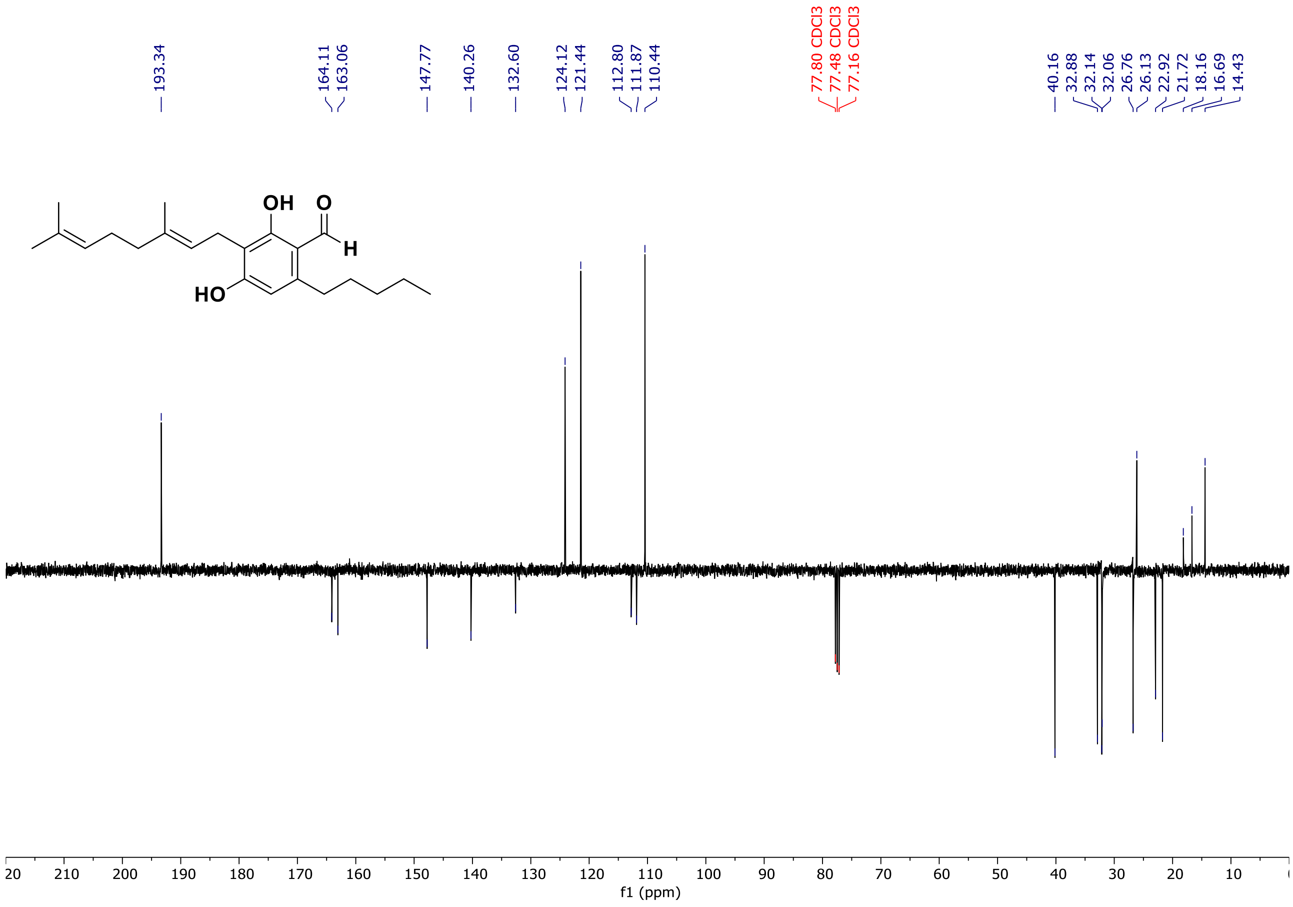

Supplementary Figure S15. APT NMR spectra (101 MHz, $CDCl_3$) of **9.**

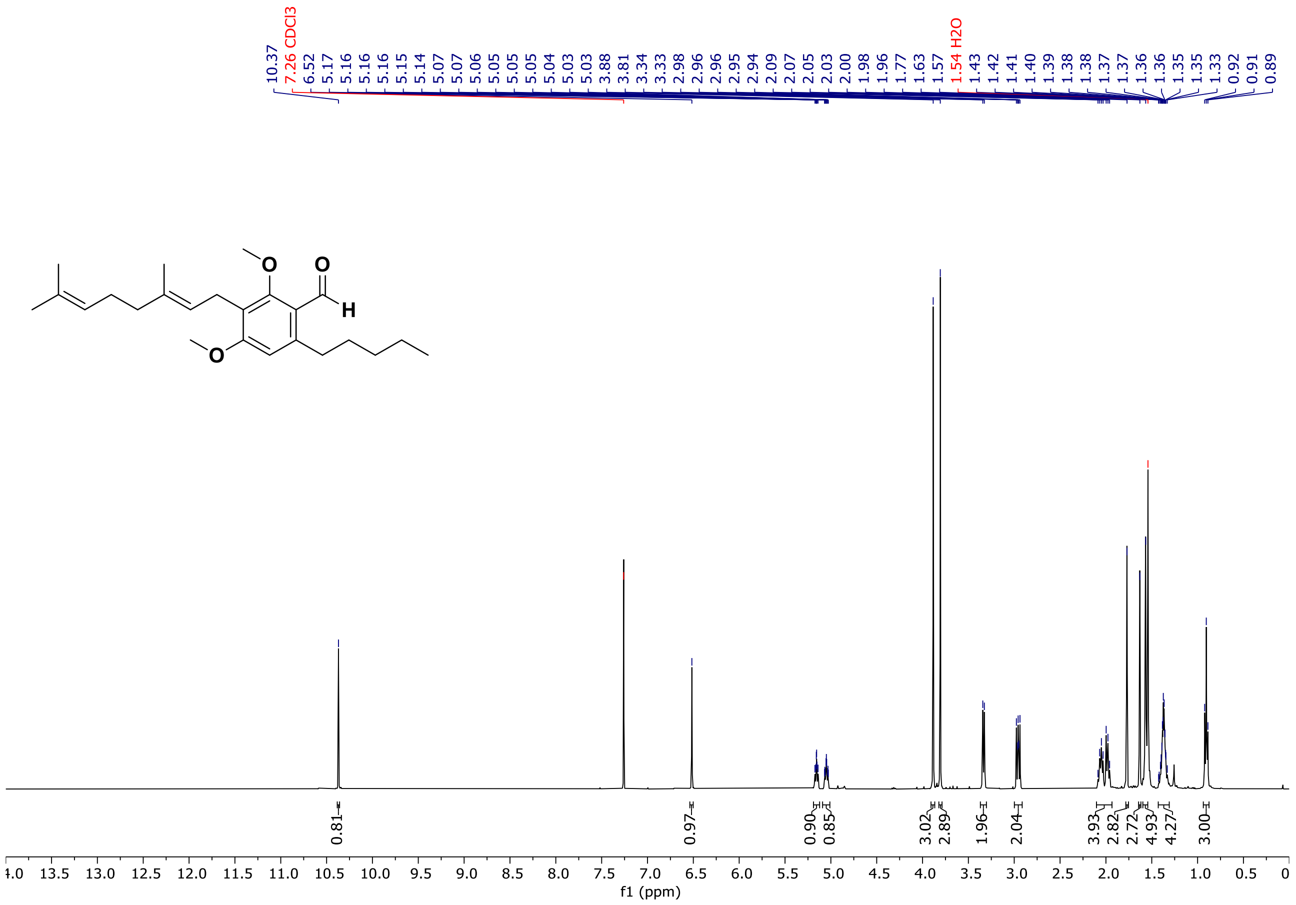

Supplementary Figure S16. ^{1}H-NMR spectra (400 MHz, $CDCl_3$) of **10.**

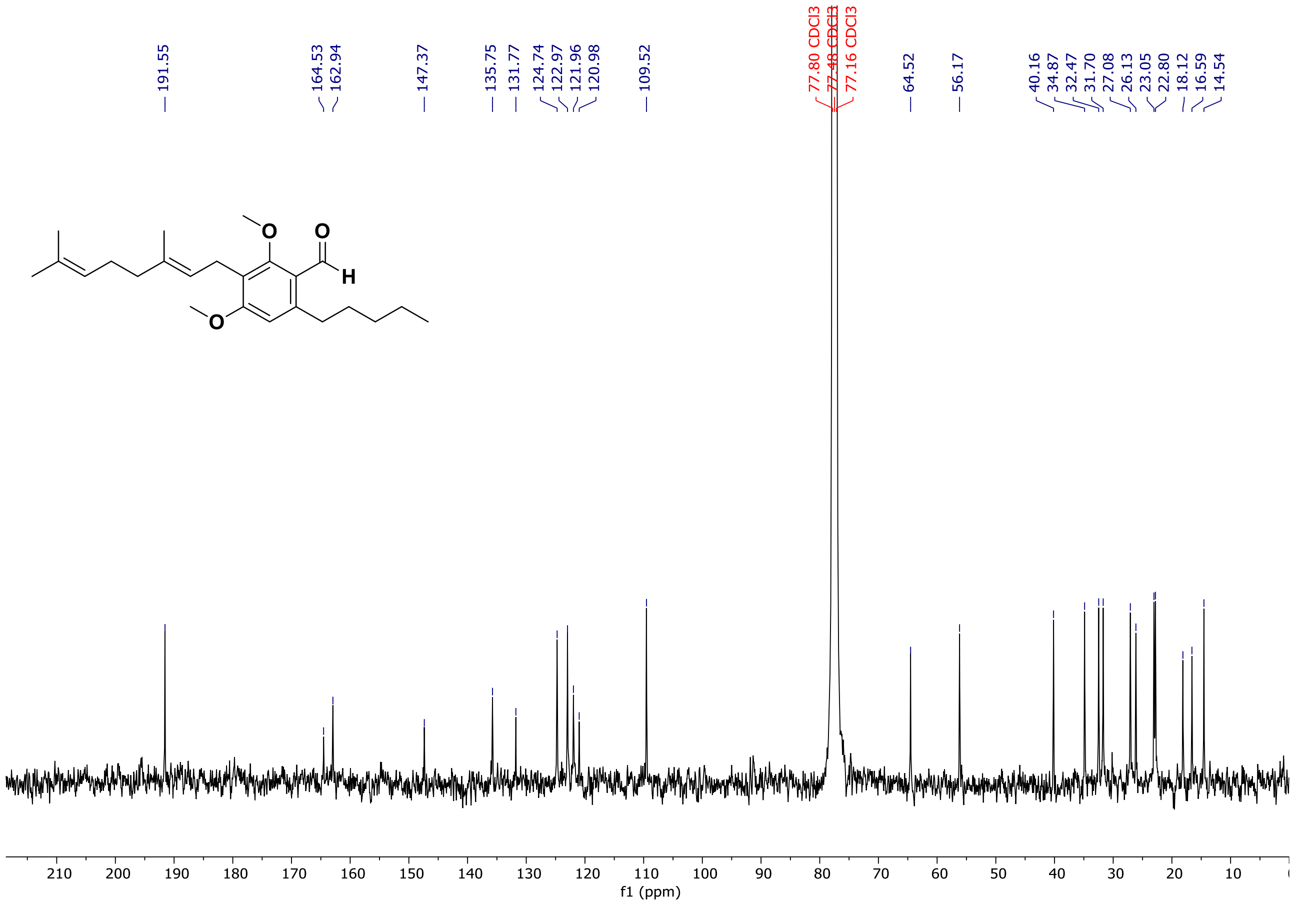

Supplementary Figure S17. ZGDC NMR spectra (101 MHz, $CDCl_3$) of **10.**

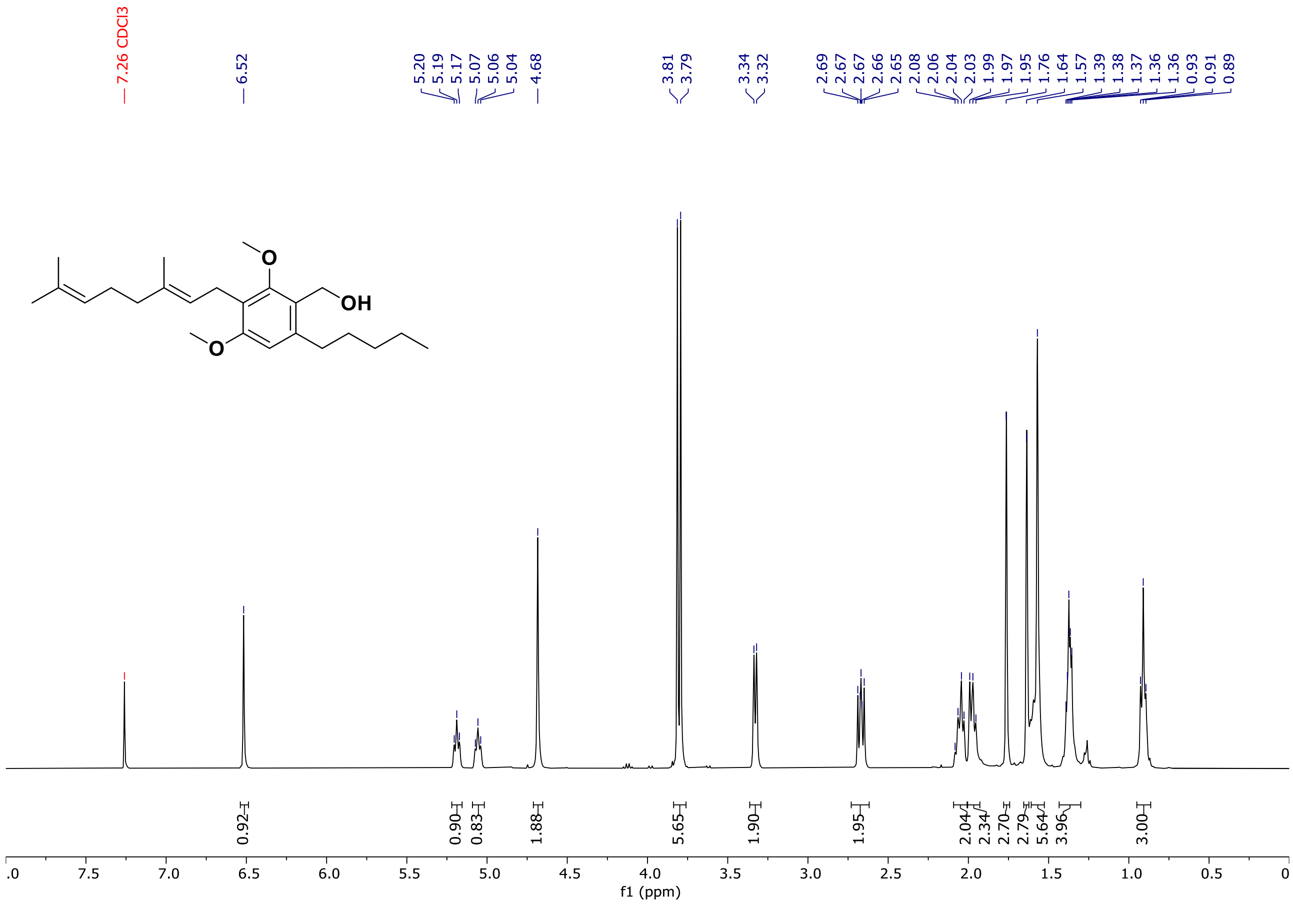

Supplementary Figure S18. ^{1}H-NMR spectra (400 MHz, $CDCl_3$) of **11.**

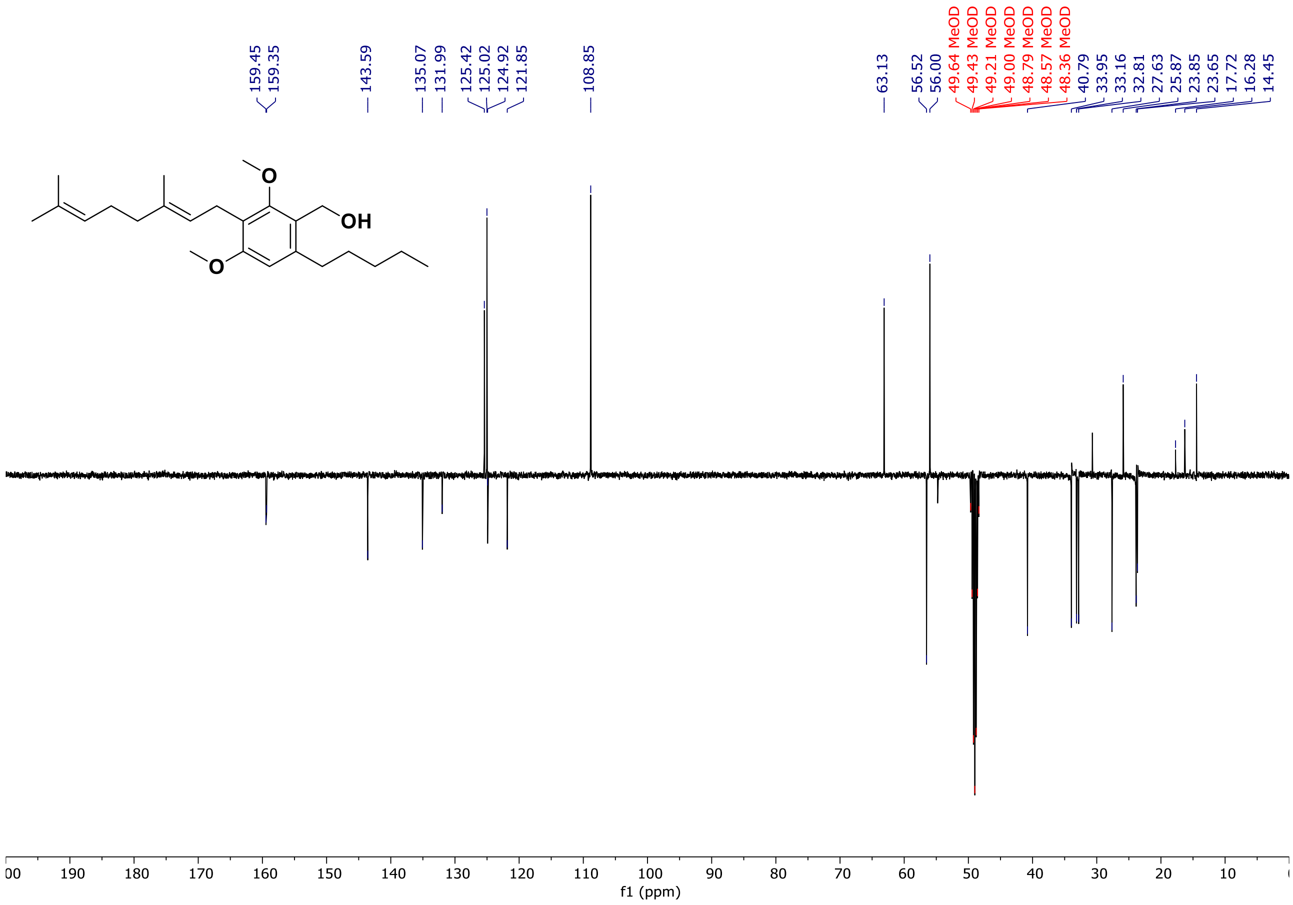

Supplementary Figure S19. APT NMR spectra (101 MHz, MeOH-d_4) of **11.**

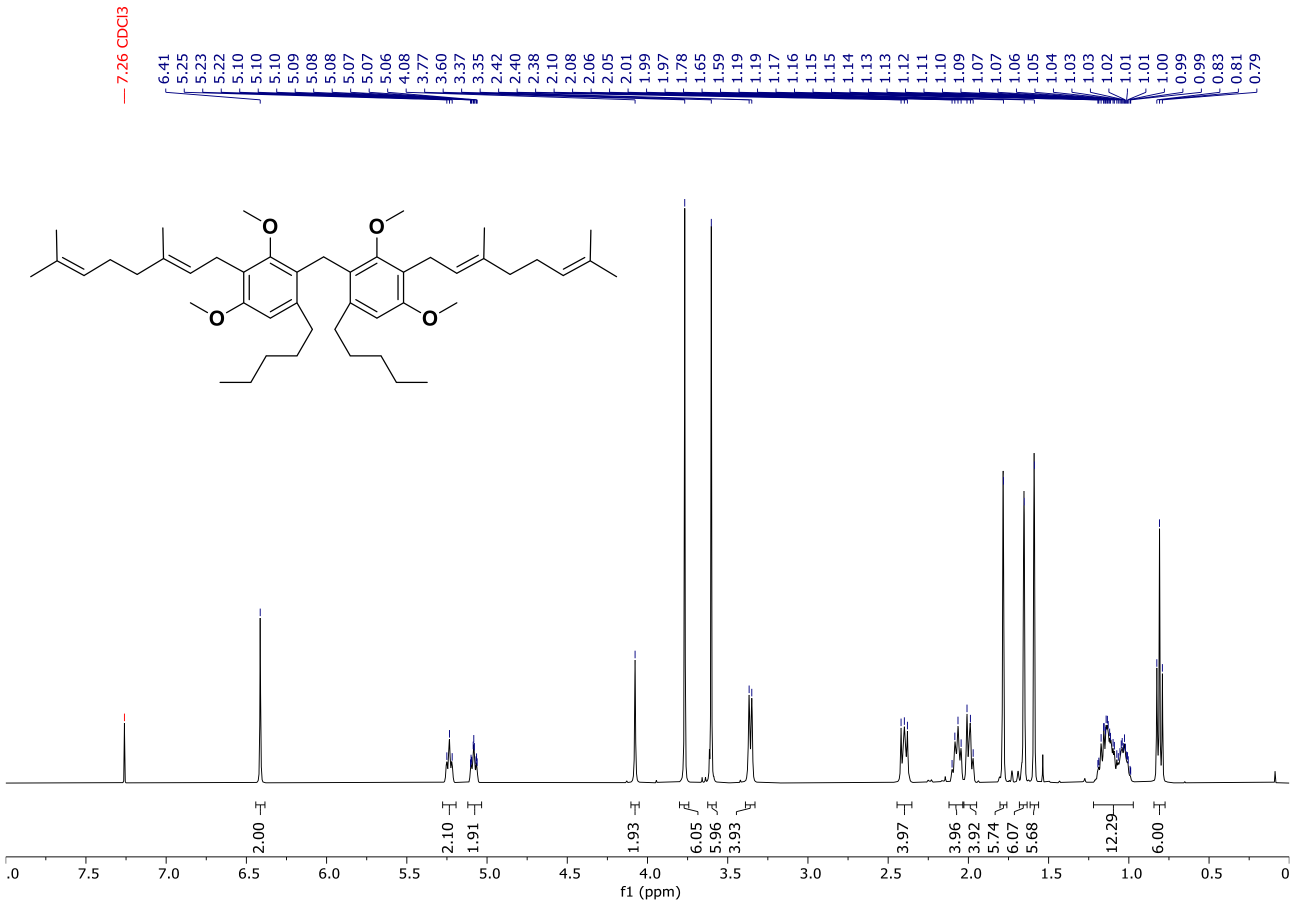

Supplementary Figure S20. ^{1}H-NMR spectra (400 MHz, $CDCl_3$) of **12.**

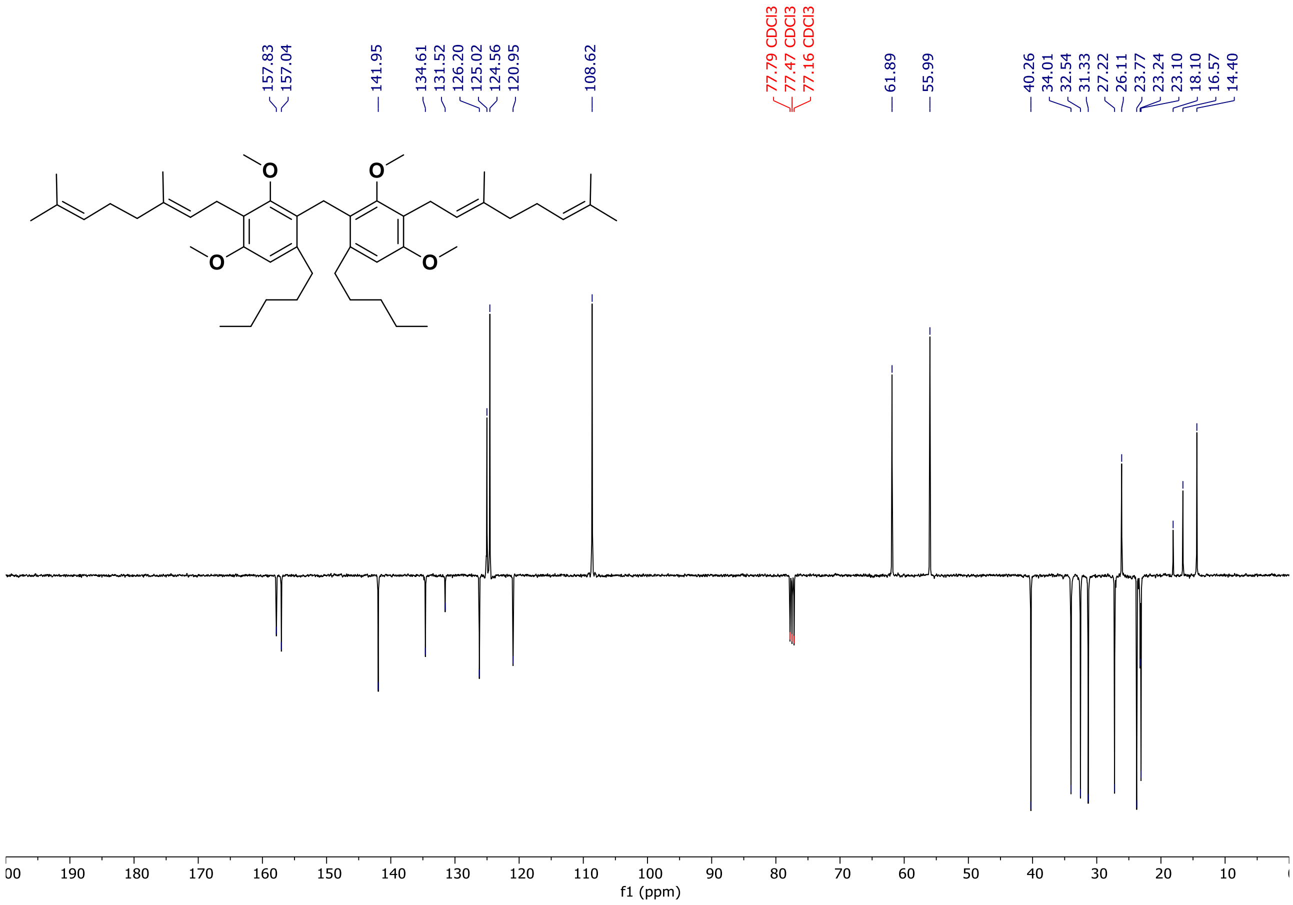

Supplementary Figure S21. APT NMR spectra (101 MHz, $CDCl_3$) of **12.**

7. HPLC traces of cannabitwinol (6) and cannabizetol (7)

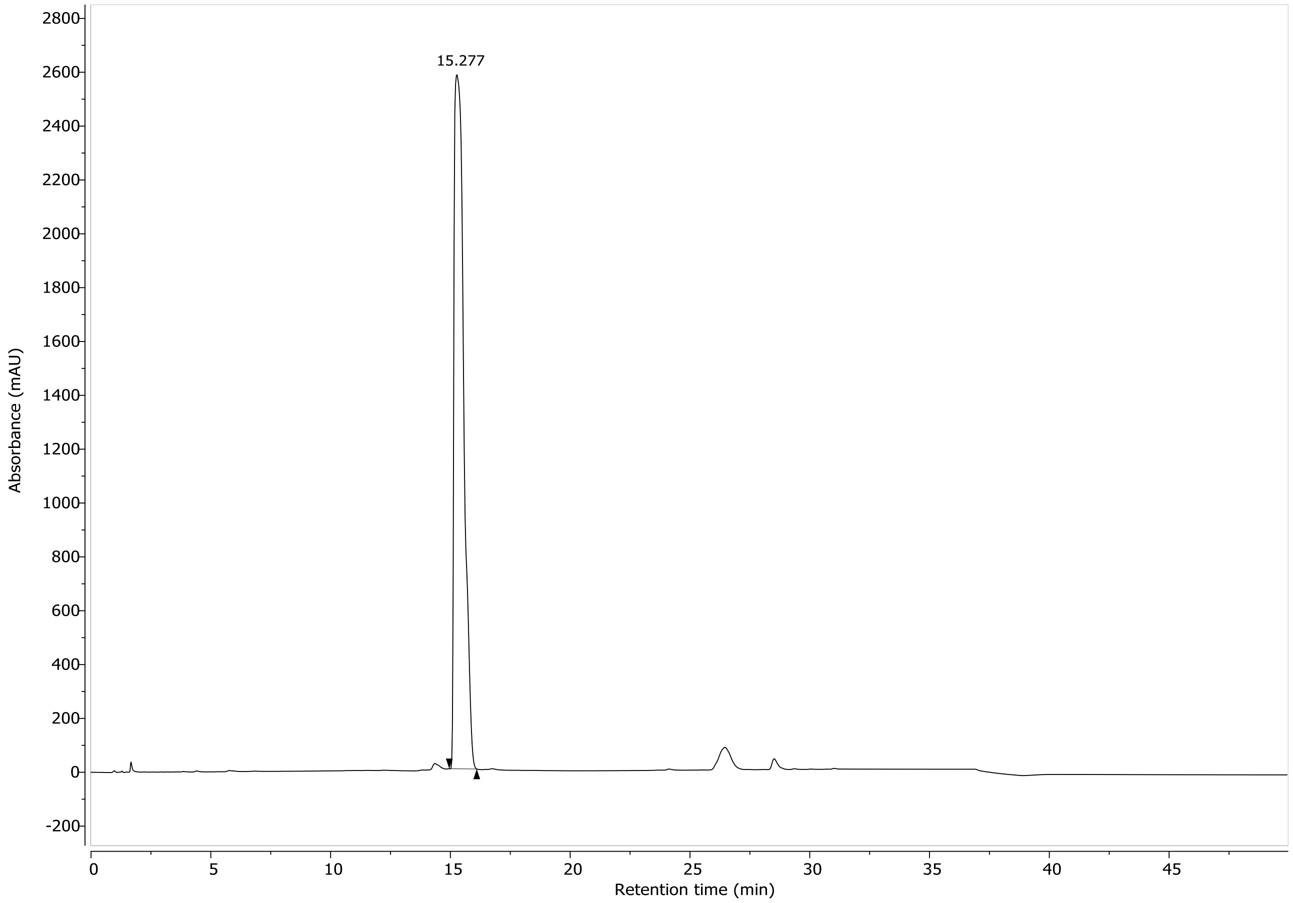

Supplementary Figure S22. HPLC chromatogram of compound **6**: Agilent 1100 Series System RP column ZORBAX SB C8 (3.5 µm x 4.6 x 150 mm), flow rate of 1.2 mL/min, UV spectra recorded at 254 nm and 220 nm with DAD detection, mobile phase H_2O/MeOH. Method: gradient for 30 min from 80% to 100% MeOH.

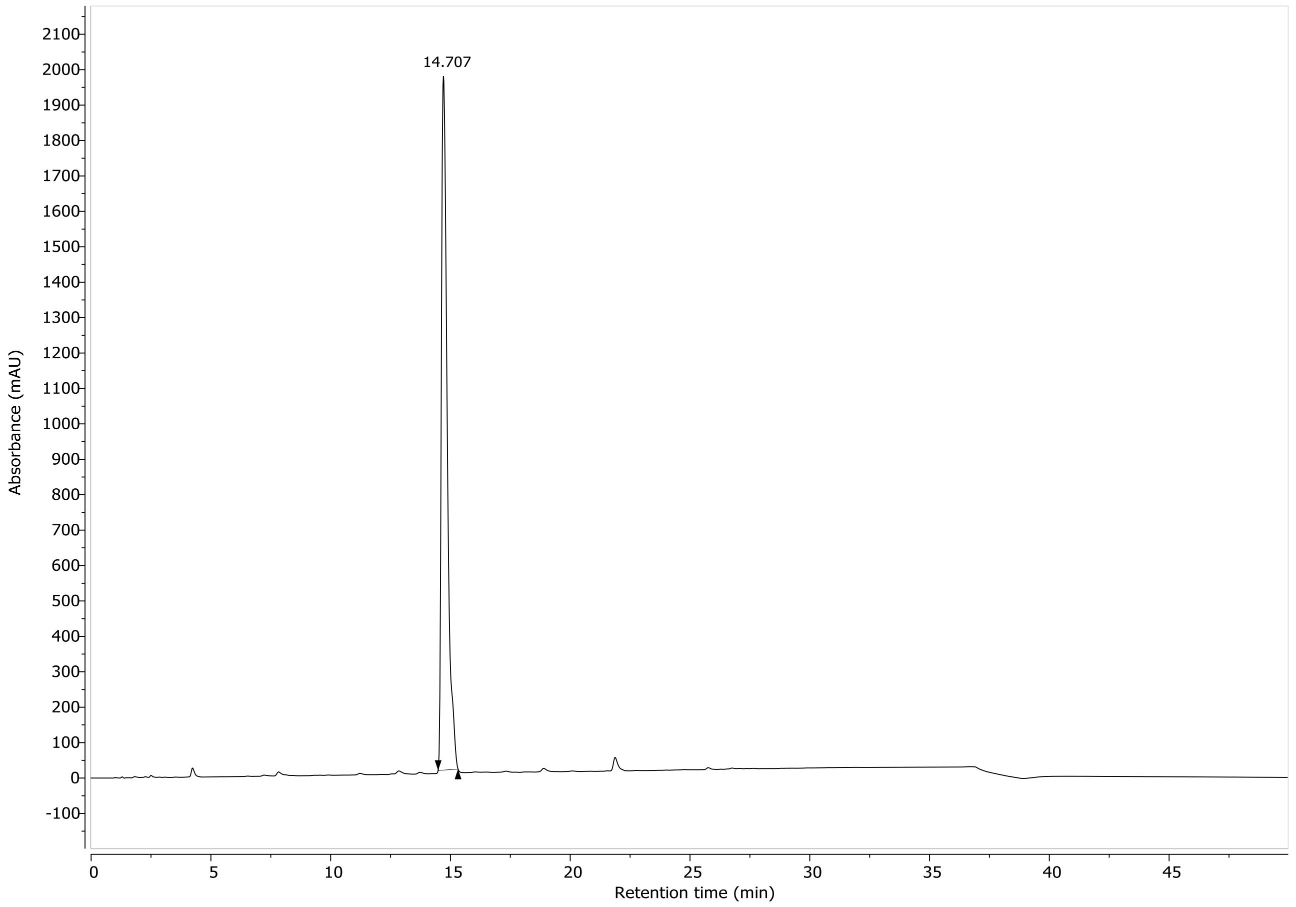

Supplementary Figure S23. HPLC chromatogram of compound **7**: Agilent 1100 Series System RP column ZORBAX SB C8 (3.5 μ m x 4.6 x 150 mm), flow rate of 1.2 mL/min, UV spectra recorded at 254 nm and 220 nm with DAD detection, mobile phase H_2O/MeOH. Method: gradient for 30 min from 80% to 100% MeOH.

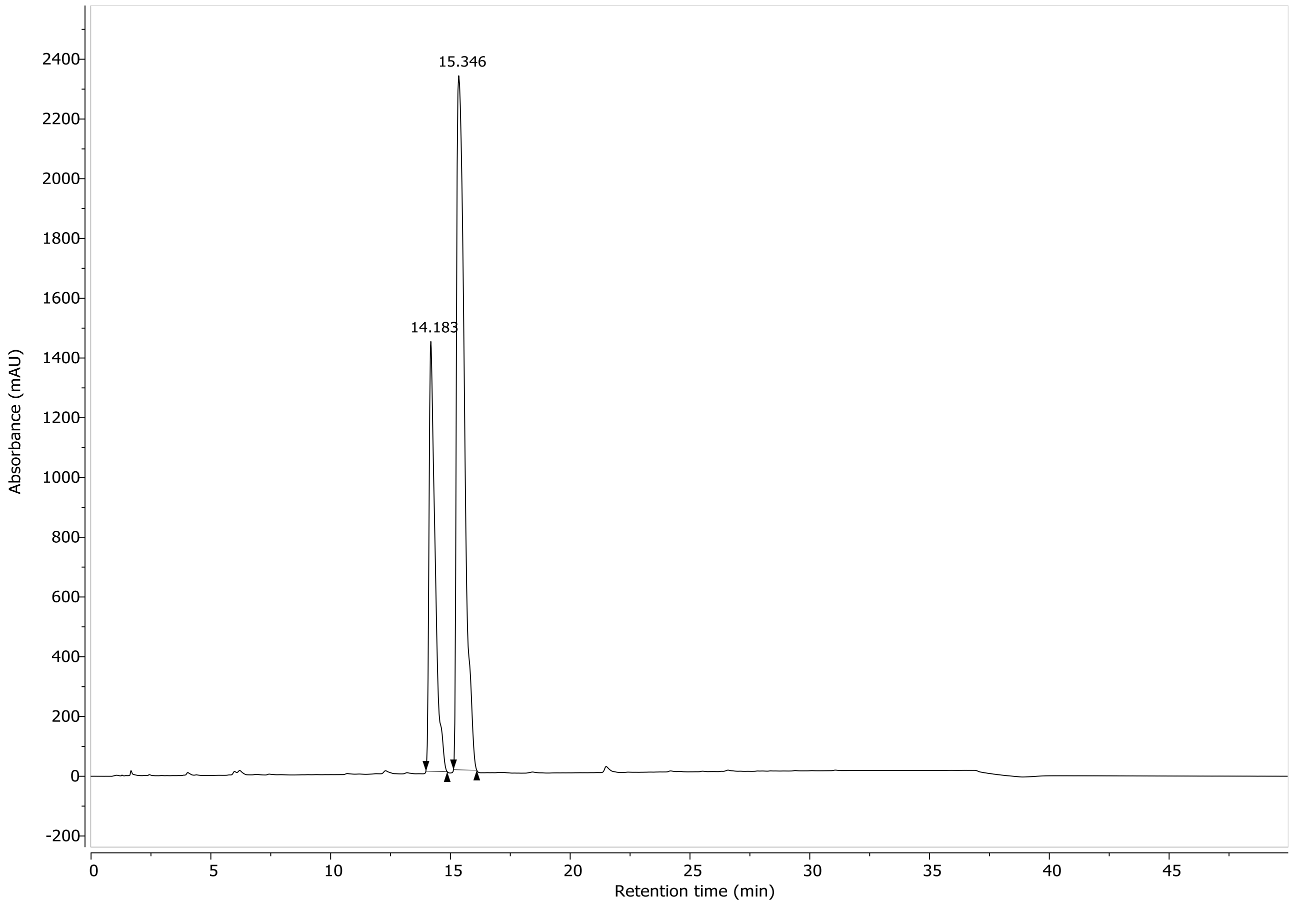

Supplementary Figure S24. HPLC chromatogram of 1:1 mixture of compound **6** and **7**: Agilent 1100 Series System RP column ZORBAX SB C8 (3.5 µ m x 4.6 x 150 mm), flow rate of 1.2 mL/min, UV spectra recorded at 254 nm and 220 nm with DAD detection, mobile phase H_2O/MeOH. Method: gradient for 30 min from 80% to 100% MeOH.

A B

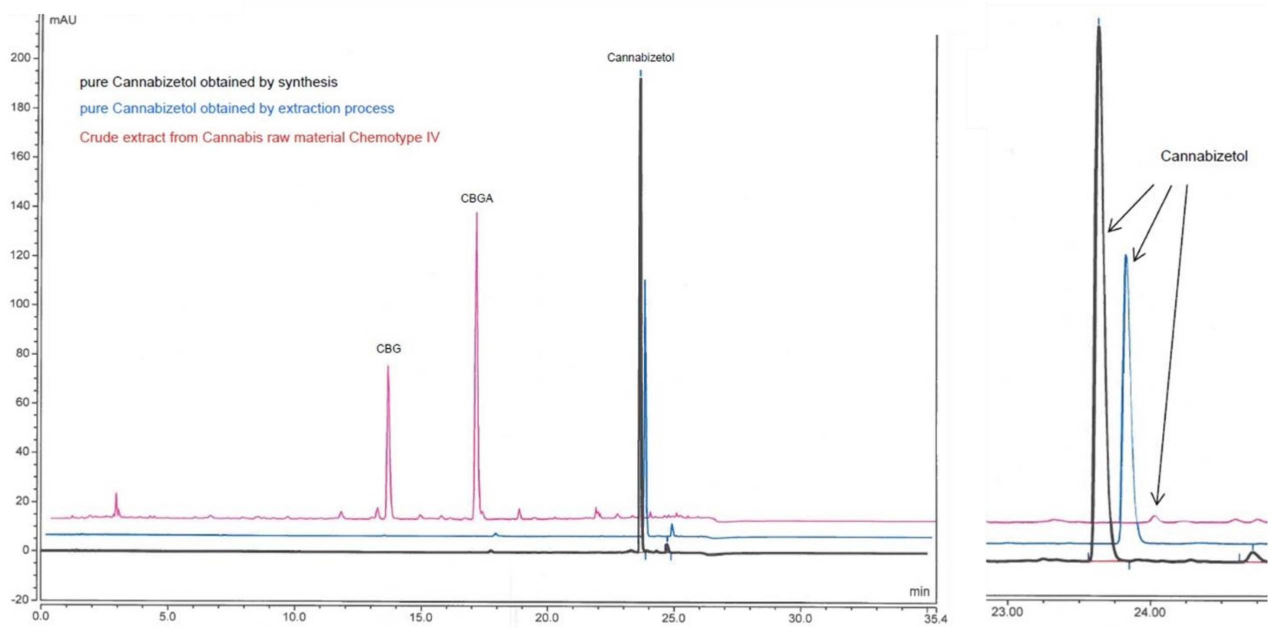

Supplementary Figure S25. Superimposition (**A**) and close up view at 24 min (**B**) of HPLC chromatograms of crude extract from Cannabis sativa raw material (in red), pure cannabizetol (**7**) obtained from extraction process (in blue) and pure cannabizetol (**7**) obtained from synthesis (in black). HPLC condition according to Ph. Eur. monograph 07/2024:3151 (Cannabidiol).
